# Cellular and Molecular Heterogeneity Associated with Vessel Formation Processes

**DOI:** 10.1155/2018/6740408

**Published:** 2018-10-10

**Authors:** Pollyana Ribeiro Castro, Alan Sales Barbosa, Jousie Michel Pereira, Hedden Ranfley, Mariane Felipetto, Carlos Alberto Xavier Gonçalves, Isabela Ribeiro Paiva, Bárbara Betônico Berg, Luciola Silva Barcelos

**Affiliations:** ^1^Department of Physiology and Biophysics, Instituto de Ciências Biológicas (ICB), Universidade Federal de Minas Gerais (UFMG), Brazil; ^2^Department of Biochemistry and Immunology, Instituto de Ciências Biológicas (ICB), Universidade Federal de Minas Gerais (UFMG), Brazil; ^3^Department of Pharmacology, Instituto de Ciências Biológicas (ICB), Universidade Federal de Minas Gerais (UFMG), Brazil

## Abstract

The microvasculature heterogeneity is a complex subject in vascular biology. The difficulty of building a dynamic and interactive view among the microenvironments, the cellular and molecular heterogeneities, and the basic aspects of the vessel formation processes make the available knowledge largely fragmented. The neovascularisation processes, termed vasculogenesis, angiogenesis, arteriogenesis, and lymphangiogenesis, are important to the formation and proper functioning of organs and tissues both in the embryo and the postnatal period. These processes are intrinsically related to microvascular cells, such as endothelial and mural cells. These cells are able to adjust their activities in response to the metabolic and physiological requirements of the tissues, by displaying a broad plasticity that results in a significant cellular and molecular heterogeneity. In this review, we intend to approach the microvasculature heterogeneity in an integrated view considering the diversity of neovascularisation processes and the cellular and molecular heterogeneity that contribute to microcirculatory homeostasis. For that, we will cover their interactions in the different blood-organ barriers and discuss how they cooperate in an integrated regulatory network that is controlled by specific molecular signatures.

## 1. Introduction

**Figure 1:**
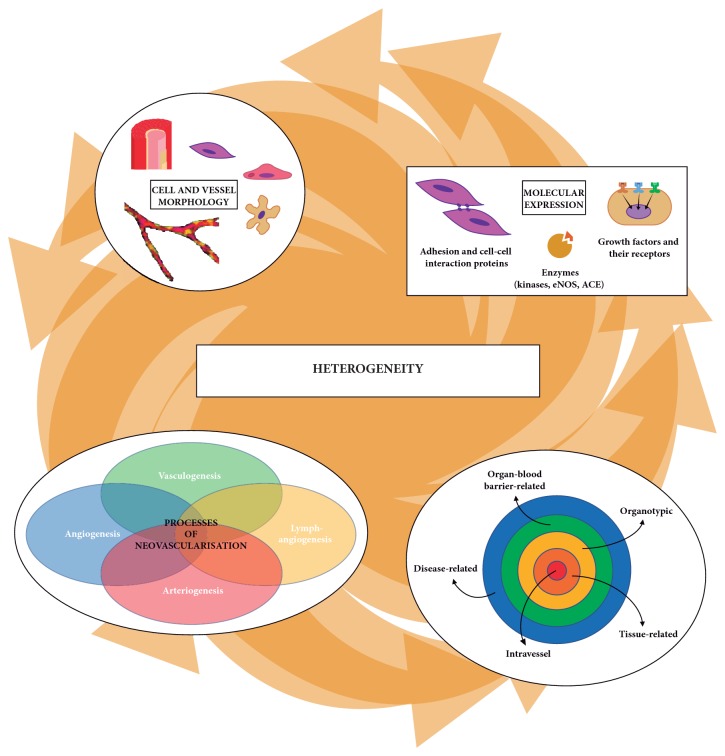
**Realms of heterogeneity in vessel formation and maintenance. **Heterogeneity can be constantly seen in the articulation of different processes of neovascularisation when building and adapting a vascular network. Those networks are site- and context-specific, with variations in the many levels of structural and functional organisation, from the systemic interaction in blood-organ barriers to intravessel diversity in cell morphology and molecular profiles and regulation, which occur both in health and disease, during embryogenesis and postnatal life. eNOS: endothelial nitric oxide synthase. ACE: angiotensin-converting enzyme. Layered macrovessel image: adapted from http://aibolita.com/sundries/12808-blood-vessel-tunics.html.

In the past few decades, much has been added to our knowledge about the diversity of structures and functions of the vascular system, especially at the microcirculation level. Undoubtedly, although a lot remains to be learned, we must be aware of the great complexity and plasticity of the microvasculature during homeostasis and scenarios of disturbance. However, the available knowledge is still largely fragmented and makes it difficult to build a dynamic view linking the microenvironments, as well as the cellular and molecular heterogeneity of blood vessels, to the basic aspects of the vessel formation processes. This review intends, therefore, to approach the aspects of microcirculation heterogeneity in an integrated way, thus allowing a broader view of how the homeostasis of the microcirculatory system is maintained (Figure 1).

A set of processes of blood and lymphatic vessel formation, here collectively assigned as neovascularisation processes, occur throughout life in both health and disease according to the functional demands of tissues. Indeed, neovascularisation is instrumental in both the formation and proper functioning of organs and systems [1, 2]. Although it is usual to study the vascular biology in a fragmented, anatomical, and/or organotypic point-of-view, the vascular network is a responsive crossing point that virtually connects all other systems and organs in the body and acts as a key player in both homeostatic and disease-progression events. Not by chance, the cardiovascular system is the first physiological system to develop in the embryo, being crucial for oxygen and nutrient delivery, as well as for waste removal and regulation of interstitial homeostasis [3].

The vascular system is known to be anatomically heterogeneous and it is essentially composed by the macrovasculature, which includes large vessels such as arteries, veins, and lymphatic vessels, that in turn branch into arterioles, venules, and capillaries, the so-called microcirculation, on which this review will be centred. Both blood and lymphatic vessels are lined by endothelial cells (EC), which are the common key cells in the main neovascularisation processes that will be addressed in this review, namely, vasculogenesis, angiogenesis, arteriogenesis, and lymphangiogenesis [4]. Of note, despite sharing a mesodermal origin and some common functions, EC are not all alike [5]. Likewise, mural cells, especially pericytes and smooth muscle cells, which will be also addressed in this review, play an important role, albeit to varying degrees, in the formation of new vessels [6, 7].

The basis of cellular heterogeneity is linked to vascular development, from embryogenesis to the formation of the mature vasculature. Mesodermal precursors, secreted by notochord during the embryonic phase in response to stimuli and factors, differentiate and originate blood islands that laterally form the primary plexus, the aorta, and the cardinal veins [8, 9]. After the maturation of vascular networks comprising arteries and veins, lymphatic endothelial cells (LEC) give rise to lymphatic vessels. Thus, the whole vascular network is developed by distinct but joint processes of neovascularisation, which are the backbone of this review [8, 10]. It is important to draw attention to the fact that vascular network formation not only precedes that of other systems and organs in the embryo but also occurs in a specialised way to meet specific demands in physiological and pathological situations throughout the (adult) life. In other words, each organ will harbour a specific vasculature depending on the stimuli to which it was submitted, leading to a tissue-specific vascular heterogeneity. Following that, in the mature vasculature, alterations on metabolic needs, interstitial fluid pressure, nutrients and oxygen availability, and shear stress are the main stimuli to generate specialised blood vessels and determine the arterial and venous fate [11–14].

In this context, we pointed out the variety of neovascularisation processes and the diversity of vascular beds, which mirrors the cellular heterogeneity of vessel walls that, in turn, echoes the molecular heterogeneity that integrates the structural and functional properties of endothelial and mural cells, associated with their homeostatic function. In this review, we intend to approach, therefore, the microvasculature heterogeneity in an integrated view considering the diversity of neovascularisation processes and the cellular and molecular heterogeneity that contribute to microcirculatory homeostasis. For that, we will cover their interactions in the different blood-organ barriers and discuss how they cooperate in an integrated regulatory network that is controlled by specific molecular signatures.

## 2. Embryo versus Postnatal Vessel Formation

Some aspects of the neovascularisation processes differ between the embryo and the postnatal period. These differences will be briefly discussed in this section before we detail each process. In the embryo, blood vessels initially form either by (1) vasculogenesis, a process by which endothelial cell precursors of mesodermal origin clump together in the so-called “blood islands” to form luminal tubes which, by invasion of tissues, extension, and interconnections, form the primordial vascular network, or by (2) angiogenesis, a process by which new blood vessels are formed from preexisting ones, promoting greater expansion and, during the phenomenon of microvascular angioadaptation, also remodelling the vascular network that matures and becomes functional [15, 16]. During the process of embryonic angiogenesis, the differentiation and specification of endothelial cells start, giving rise to the arterial and venous systems [17]. Therefore, the process of defining the cellular identities linked to the different vascular beds begins, leading to the establishment of the primary phenotypic heterogeneity of the cells composing vessel walls. Importantly, later in embryogenesis, after the onset of heart beats and blood flow, (3) arteriogenesis takes place as a process by which arterial branches are formed through remodelling of preexisting arterioles, primarily driven by hemodynamic forces [18–20]. Finally, following venous morphogenesis, lymphatic vasculature begins to emerge from precursors derived from the cardinal vein [21] or the yolk sac [22], then initiating the process of (4) lymphangiogenesis [23].

Postnatal neovascularisation, although much more related to the microvasculature, is represented by a greater variety of events of blood vessel formation when compared to the embryo, especially if we consider that, in addition to the physiological situations, the organisms are also exposed to a range of disruptive situations such as inflammatory events, ischaemic and/or tumoural disorders, which go far beyond simple genetic programming. Regarding the neovascularisation processes, we may highlight that (a) postnatal vasculogenesis, unlike its embryonic counterpart, mostly supports (b) the angiogenic process (which is, in fact, the predominant neovascularisation process in adults), as well as small endothelial repairs [24, 25]; (c) arteriogenesis, being responsible for either* de novo* growth or remodelling of preexisting collateral arterial branches, guides the redistribution of the blood flow from blocked arteries to ischaemic areas [26]; (d) lymphangiogenesis plays an important role during inflammatory responses and the elaboration of specific immune responses, no longer reducing lymphatic capillaries to a merely accessory maintenance pathway of interstitial fluid homeostasis [27, 28]; and (e) vascular mimicry occurs through differentiation of tumour stem cells and/or dedifferentiation of mature tumour cells into cells that line up in tubular structures to increase blood supply during the development of highly invasive cancers [29, 30].

## 3. Diversity of Neovascularisation Processes

**Figure 2:**
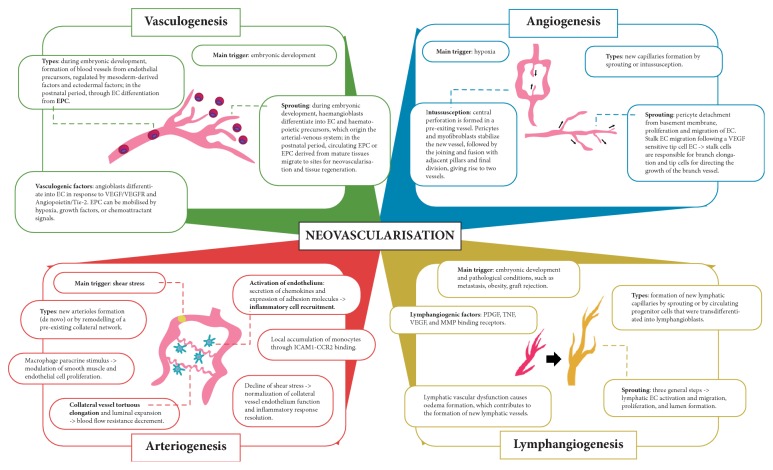
**Heterogeneity among the processes of neovascularisation. **Principles of the different processes regarding their triggers, phases, protagonist cells, and molecular profiles. EC: endothelial cell. EPC: endothelial progenitor cell. VEGF: vascular endothelial growth factor. VEGFR: VEGF receptor. Tie-2: angiopoietin-1 receptor. ICAM1: intercellular adhesion molecule 1. CCR2: C-C chemokine receptor type 2. PDGF: platelet-derived growth factor. TNF: tumour necrosis factor. MMP: matrix metalloproteinase.

According to the previous topic, different neovascularisation processes may predominantly occur at different stages of development. Thus, during embryogenesis, vascular network formation occurs initially by the process of vasculogenesis followed by expansion by angiogenesis in blood vessels and lymphangiogenesis in lymphatic vessels. In the postembryonic period, the predominance of processes such as angiogenesis and arteriogenesis is observed, although the occurrence of vasculogenesis and lymphangiogenesis has been discussed more recently. Moreover, the heterogeneity of processes is not limited to the interprocess level, but is also seen within the same neovascularisation process. This section will discuss the different processes of neovascularisation, as well as the existing diversity among them and within the same type of vascular formation, here named intraprocess heterogeneity, regarding how they occur, the cellular and molecular factors involved, and their characteristics in physiological and pathological conditions (Figure 2).

### 3.1. Vasculogenesis

Vasculogenesis, the formation of new blood vessels from endothelial precursors, occurs in embryo and conceivably in adults. Embryonic vasculogenesis initiates in the early stages of development from mesodermal cells and is known to be positively regulated by mesoderm-derived factors and negatively regulated by ectodermal factors [31, 32]. Postnatal vasculogenesis, in turn, is supposed to occur during endothelial cell differentiation from putative endothelial progenitor cells (EPC) that may be derived from the bone marrow, vascular endothelium, or other mature tissues [33–36].

In the embryo, vasculogenesis results from the differentiation, expansion, and coalescence of mesoderm-derived CD34^+^ haemangioblasts. These cells are first established in the extraembryonic yolk-sac mesoderm followed by the emergence in the intraembryonic mesoderm and, then, converge in an initial vascular network, named primary vascular plexus, which originates from the artery-venous system [37–41]. Driven by Ets-related protein 71 (ER71), GATA binding protein 2 (GATA-2), and stem cell leukaemia (SCL), haemangioblasts are able to differentiate into angioblasts and haematopoietic precursors [42]. Angioblasts continue to differentiate into endothelial cells especially in response to vascular endothelial growth factor (VEGF)/VEGF receptor and angiopoietin/Tie-2 pathways [43–46]. The newly formed blood vessels, besides supplying nutrients and oxygen to the growing tissues, are also a source of factors and trophic signals that guide organogenesis [47].

On the contrary to the common sense recognised since the beginning of the 20th century, Asahara and colleagues [48] suggested the existence of circulating angioblasts not only in embryos, but also in adults, and then postulated the existence of postnatal vasculogenesis. In fact, the so-called circulating EPC were shown to migrate into ischaemic and wound healing sites where new blood vessels are needed to support tissue survival and/or (re)establishment [49, 50]. In fact, putative EPC are known to play an important role in neovascularisation, tissue regeneration, and organogenesis [51].

*In vitro* assays initially qualified circulating putative EPC into two distinct populations: (a) early outgrowth cells, which raise from haematopoietic-derived small colonies of cells with no/low proliferative activity, and (b) late outgrowth cells, also known as endothelial cell forming colony (ECFC), that have a high proliferative capacity. Currently, however, the early “EPC” are more appropriately referred to as circulating angiogenic cells (CAC) and have been shown to be a monocyte/macrophage lineage derivative [52–55]. Likewise, markers for human putative EPC, such as CD34, fetal liver kinase 1 (FLK-1)/VEGFR2, and Tie-2, that have been regularly referred to in the literature may also indicate cells of haematopoietic lineage because these molecules are commonly shared among them.

Although it was believed that the bone marrow was the main source of putative EPC in the adult, evidence suggests the presence of a hierarchy of vascular progenitor cells in the endothelium that can participate in the neovascularisation processes [56]. Recently, Fang and colleagues [35] elegantly demonstrated that the endothelium may represent an important and neglected source of EPC. In their study, the so-called vascular endothelium-resident stem cells (VESC) are shown to display high clonogenic capacity and are positive for CD117/c-Kit. Moreover, these authors showed that the absence of CD117 impairs the endothelial maturation of VESC. In fact, these data, along with those of other subsequent studies on VESC [57], have made it possible to construct a new paradigm which postulates that EPC derived from the endothelium have a greater proliferative capacity and are able to integrate new growing vessels, whereas bone marrow-derived “EPC” act in a paracrine manner and are actually haematopoietic cells supporting angiogenesis.

In addition, a side population of vascular progenitors can be isolated from the tunica media of the aorta. When cultured in the presence of VEGF or transforming growth factor (TGF)-*β*1 and platelet-derived growth factor subunit B (PDGF-B), they originate cells with endothelial (positive for CD31, VE-cadherin, and von Willebrand factor) or smooth muscle phenotypes (positive for *α*-smooth muscle actin, calponin, and smooth muscle myosin heavy chain) [58]. These progenitor cells have a Lin^−^Sca-1^+^c-Kit^-/low^CD34^-/low^ profile and do not form myeloid or lymphoid colonies. Zengin and colleagues [59] suggested coining the structure located between the smooth muscle and the adventitious layer of large and medium vessels as “vasculogenic zone”. This region would be characterized by cells expressing VEGFR-2 and Tie-2, but negative for CD31, CD45, and CD146. The clonogenic and proangiogenic capacity of these adventitious progenitor cells (APC) was demonstrated, as well as their ability to express both pericyte (neuron-glia antigen 2—NG2—and PDGF receptor *β*) and mesenchymal cells markers (CD44, CD90, CD73, and CD29) [60–62].

Currently, there is a general understanding that cells from myeloid (and lymphoid) lineage, which are derived from bone marrow, and circulating truly EC precursors, which may be derived from the blood vasculature, are, respectively, able to offer angiogenic support or differentiate into EC, giving rise to new blood vessels in adults [35, 53, 63–67]. In fact, adult putative EPC may be responsible for the neo-endothelialisation and postnatal vascularisation, not exclusively by vasculogenesis, but mainly by assisting angiogenesis [68]. In addition, EC precursors seem to be present in other tissues and may display different phenotypes depending on the organ of origin [36, 69]. Lastly, similarly to haematopoietic stem cells (HSC), putative EPC may home and populate the spleen in response to chemical stimuli, inflammation, and neoplasia, for example [70–72].

Overall, EPC are assumed to be mobilised and migrate to where new blood vessels are needed and they may either differentiate into EC and be incorporated into the growing vessel or exert paracrine functions, therefore, combining proper vasculogenic and supportive angiogenic roles, respectively [68, 73]. EPC mobilising signals may vary from hypoxia, growth factors, to chemoattractant signals [25]. In fact, growth factors such as VEGF, granulocyte-macrophage colony-stimulating factor (GM-CSF), fibroblast growth factor (FGF), and insulin growth factor 1 (IGF-1) are shown to promote both mobilisation and differentiation of CD34^+^/VEGFR-2^+^ cells [74, 75].

In physiological conditions, circulating EPC are present in a very reduced number in the peripheral blood. Nevertheless, in situations of tissue hypoxia, for example, there is an increase in the production of EPC mobilising factors. These factors may activate endothelial nitric oxide synthase (eNOS), increasing the production of nitric oxide (NO), which regulates the enzymatic activity of matrix metalloproteinases (MMP). Particularly, MMP-9 leads to the release of soluble kit ligand (sKitL) from EPC surface, resulting in the mobilisation of these cells from bone marrow niches to the peripheral circulation [76, 77].

The capacity of EPC to generate new blood vessels is associated with their heterogeneity and phenotype. Also, the genetic background may account for that, once the basal circulating number of EPC may vary in different mouse strains [78] and it is associated with the ability of these cells to respond to angiogenic stimuli, such as FGF or VEGF, in different vascular beds [79, 80]. Thus, heterogeneity could extend within the organ itself and its vascular segment due to differences in the physical and chemical environment. In this sense, some organs present their primary vascularisation focused exclusively on vasculogenesis such as the lung, while other organs such as the kidneys are vascularised by both vasculogenesis and angiogenesis. Indeed, it is proposed that embryonic leaflets are associated with distinct neovascularisation processes, where organs from endodermal origin are vascularised by vasculogenesis and ectodermal organs by angiogenesis [81].

In a pathological context, vasculogenesis is important during tumoural development, especially when angiogenesis is inhibited by antitumoural therapies. An increase in circulating EPC populations has been shown in patients with cancer, suggesting a possible role of vasculogenesis during tumoural neovascularisation [82]. Furthermore, several studies have been showing a correlation between the reduction of EPC and worsening of reendothelialisation in injured vessels, metabolic diseases, diabetes, atherosclerosis, and endothelial dysfunction [83].

### 3.2. Angiogenesis

Angiogenesis, differently from vasculogenesis, is the process by which new blood vessels are formed from preexisting vessels. It is relevant in both physiological and pathological conditions, occurring by two different mechanisms: sprouting and intussusception.

Sprouting angiogenesis is the formation of new blood vessels in response to proangiogenic factors and involves retraction of pericytes, migration, and proliferation of EC that give origin to branches with a lumen, which are stabilised by pericyte recruitment and deposition of a new basement membrane [84]. This process is initiated by tissue hypoxia and it is seen occurring in physiological processes such as embryonic development, particularly important for the vascular network expansion initially formed by the vasculogenesis, in the wound healing process, during ischaemic tissue vascularisation, and in pathological conditions, such as solid tumours formation, eye diseases, and inflammatory disorders as rheumatoid arthritis and psoriasis [85–88].

In turn, intussusception angiogenesis consists in the repeated insertion of new, slender, transcapillary tissue pillars, which increase in size and allow capillary network growth [89]. This angiogenesis type is more efficient compared to sprouting since it only requires a reorganisation of EC without initial cell migration and proliferation, being, therefore, an economic process of neovascularisation regarding metabolic and energetic demands. Intussusception occurs throughout life but is more significant during vascular development in embryos; however it also may occur from preexistent capillaries and the current methods for their evaluation remain failed and expensive, which limits the advances and knowledge about this type of angiogenesis [90, 91]. The existence of these two types of angiogenesis suggests a form of heterogeneity regarding the cells, factors, and stimulus among them. In addition, the preference for one or another process indicates the importance of each type of angiogenesis in a particular condition or stage of embryonic development. Despite this hypothesis, more studies are necessary to address the factors and conditions determining the heterogeneity during sprouting and intussusceptive angiogenesis in different blood-organ barriers and tissues.

Angiogenesis process is controlled by a range of angiogenic stimulators and inhibitors, on a way that the balance of these factors maintains the turnover of endothelial cells. However, under conditions such as reduced pO_2_, low pH, hypoglycaemia, mechanic stress, inflammatory stimuli, and tumoural development, there is an increase of proangiogenic factors, inducing endothelial proliferation, and migration, triggering a so-called angiogenic switch [74, 92]. In general, the factors involved in the angiogenic process are VEGF and hypoxia-inducible factor 1 (HIF-1), the main agents that orchestrate vascular homeostasis and promote an increment on vascular permeability, migration, and proliferation of EC [88, 93]; angiopoietin-1 and angiopoietin- 2 (Ang-1 and Ang-2), which exert antagonistic functions during vessel development, since Ang-1 inhibits vascular permeability, whereas Ang-2 is involved in vessel destabilisation by detachment of microvascular mural cells (MMC) [74, 94]; matrix metalloproteinases (MMP), which enhance angiogenesis through the degradation of matrix components [95]; integrins* αβ*, which help EC migrate by promoting EC adhesion to vessels [96, 97]; and PDGF-BB and PDGFR, which participate in blood vessel maturation [98, 99].

Pathological conditions induce the activation of different molecular pathways during angiogenesis when compared to physiological states. Mutations in oncogenes and tumour suppressor genes, and growth factor misbalanced activities are crucial to trigger pathological angiogenesis. Physiological angiogenesis is dependent on VEGF and HIF-1 family, whereas, in tumoural angiogenesis, there is a recruitment of myeloid cells and an upregulation of alternative vascular growth factors such as PIGF and FGF, besides VEGF releasing [87].

In sprouting angiogenesis, EC present three distinct phenotypes which play different roles in the sprouting vessel, named angiogenic tip, stalk, and phalanx cells. Specialised tip cells are found driving sprouting vessels and present an important and characteristic delta-like 4 (DLL-4)/NOTCH-1 signalling, which suppress tip cell fate in the neighbouring EC. In addition, they express C-X-C chemokine receptor type 4 (CXCR-4), netrin receptor UNC5B, NRP1, and secrete ligands that act on stalk cells adjacent such as Ang-2 [100, 101]. In relation to their morphology, tip cells show organised stress fibres with numerous probing filopodia that permit migrating toward angiogenic factors, but they do not form lumen and are minimally proliferative [102, 103]. Stalk cells are seen behind tip cells and are highly proliferative, form lumens, and lay down extracellular matrix, but do not extend filopodia. In addition, stalk cells show attenuated DLL-4 expression due to an inhibition promoted by NOTCH signalling from adjacent tip cells, while they present Ang-2 receptor and Tie-2, two components not present in tip cells [100, 104, 105]. Phalanx cells are morphologically cobblestone-shaped and have lower migratory and proliferative capacities, but present high expression of VEGFR-1, which is responsible for interposing the proangiogenic effects of VEGF and keeping phalanx cells in a quiescent state, together with a high expression of VE-cadherin. Thus, phalanx cells seem to respond to VEGF in a different way than tip cells [103, 106].

Under physiological conditions, EC in the blood form a monolayer of phalanx cells interconnected by junctional molecules ensheathed by pericytes, which suppress EC proliferation. In sprouting angiogenesis, when proangiogenic signals such as VEGF, FGF, or chemokines are released by any of the aforementioned stimuli, pericytes detach and liberate themselves from the basement membrane by proteolytic degradation, mainly by MMP [107]. At the same time, VEGF increases the permeability of the vessel and plasma proteins extravasate, forming a provisional extracellular matrix (ECM) on which EC migrate and initiate branch formation. Some EC are more responsive to VEGF and present a migratory behaviour due to a particular and specific gene expression profile that includes VEGFR-2 high expression, acquiring a tip cell phenotype. They are able to integrate attractive and repulsive directional cues presented by the microenvironment and define the route in which the new sprouts grow [104, 108]. In addition, tip cells activate NOTCH signalling in adjacent EC, initiating lateral inhibition and inducing a “stalk cell” profile in these adjacent cells, indicating the importance of cellular heterogeneity in the sprouting angiogenesis process [109, 110]. The proliferation of stalk cells is responsible by branch elongation, while tip cells direct the growth of the branch vessel by filopodia and lamellipodia [111, 112]. Guidance receptors are expressed by tip cells, such as ROBO4, UNC5b, PLEXIN-D1, NRPS, and Eph family members which are able to probe the microenvironment and play action that culminates in maintenance of vessel integrity and directed migration by filopodia and lamellipodia promotion [113, 114]. Tip cell fusion and branch anastomosis are facilitated by macrophages that express angiopoietin receptor TIE-2, NRP1 receptor (a specific receptor for semaphorins), and VEGF, acting to modulate intercellular adhesion [115]. In addition, to support this idea, Fantin and colleagues [116] demonstrated that macrophages expressing TIE-2 and NRP1 comprised the major population of tissue macrophages acting on brain vascularisation and they were able to interact with tip cells, promoting vascular anastomosis and indicating a new target to antiangiogenic therapies.

Finally, a lumen is formed by one of three processes: intracellular vacuole coalescence, intercellular vacuole exocytosis, or luminal repulsion, leading to subsequent relocalisation of junctional proteins to the lateral membranes and giving rise to a new blood vessel [117]. Mural cells such as pericytes and smooth muscle cells are recruited and promote stabilisation of the new vessel while parallel matrix deposition and specific vascular bed adaptations occur [104, 118, 119]. Vessel perfusion is initiated in response to prolyl hydroxylase domain protein 2 (PHD2) inactivation by hypoxia, which leads to increased expression of hypoxia-inducible factor 2*α* (HIF-2*α*), triggering VEGFR-1 and VE-cadherin expression, essential to normalisation of EC function and vessel perfusion [109].

In an intussusceptive angiogenesis process, the new blood vessel is formed from a preexisting vessel following an intraluminal pillar formation and interendothelial reorganisation. Then, a central perforation is formed and pericytes and myofibroblasts stabilise the new vessel, a step followed by the joining and fusion with adjacent pillars and final division, giving rise to two vessels [120, 121]. This process shows three main patterns of vascularisation: (1) intussusceptive microvascular growth (IMG), which occurs by a fast expansion of the vascular network and is directed by blood flow requirement, forming the organ-specific architecture; (2) intussusceptive arborisation that is implied in a serried pillar remodelling in a disorganised vascular network like a typical tree-like arrangement and is not seen in a hierarchical pattern; and (3) intussusceptive branching remodelling, which seems to occur close to vascular bifurcations and is important to promote an optimisation of blood vessel number [120–122].

The intussusceptive angiogenesis mechanism is important to growth and vascular remodelling, besides being present in pathological contexts such as tumour development. The regulators in this process are hemodynamic forces, blood flow, and soluble factors, among which are VEGF, FGF, PDGF, angiopoietins, and HIF [120]. Few experimental models are available to study mechanisms, factors, and in which conditions intussusceptive angiogenesis occurs, since it is occurring in an intravascular compartment. Therefore, the discovery of new experimental approaches is required to elucidate this neovascularisation process.

In a recent research, however, Hlushchuk and colleagues [123] demonstrated that a downregulation of endoglin (ENG/CD105) with an upregulation of chicken ovalbumin upstream promoter transcription factor II (COUP-TFII) was implicated in intussusceptive microvascular growth, correlated with pillar formation increment in models of intussusceptive angiogenesis in chicken embryos and acute glomerulonephritis in rats [123]. De Paepe and colleagues [124] described an aberrant nonsprouting angiogenesis in human fetal lung xenografts that was associated with bronchopulmonary dysplasia of preterm newborn (BPD)-associated dysangiogenesis. In this situation, lungs are able to change vascular pattern formation from a sprouting phenotype to an intussusceptive phenotype, showing a linear and nonsprouting vasculature probably due a mechanism that includes IGF signalling dysregulation and hypoxia, not yet clarified. In addition, a proper formation of the pulmonary microvasculature seems to be necessary to the normal alveolar and lung development [124–126].

In addition to the heterogeneity of EC and angiogenic processes, in many conditions such as tumourigenesis, the formed vascular bed varies considerably depending on the type and site of the tumour. This heterogeneity could explain, at least in part, the resistance of some tumour types to antiangiogenic therapies [127]. In general, the tumour vasculature is characterized by exacerbated angiogenesis and abnormal tortuous blood vessels, discontinuous EC, and scarce pericyte coverage, which favour the extravasation of tumour cells [73, 128]. EC from tumours differ from healthy EC for a range of reasons, but mainly due to their expression of a subset of genes that could vary depending on tumour type, exhibition of a proangiogenic and stem-like phenotype, and chromosomal abnormalities [129, 130]. All these peculiarities contribute to the transformation of tumour angiogenesis in a complex process and, consequently, foment discussions about angiogenic modulation in relation to tissue heterogeneity, especially with respect to therapies and the advent of new imaging tools for diagnostic purposes. It has already been observed that the density of the initial microvasculature in the primary tumour site is determinant to tumoural progression and further neovascularisation.

In conclusion, angiogenesis is characterized by a heterogeneous and complex process which includes a range of factors worthy of being subject of studies for future applications. Furthermore, these complexities, especially regarding cellular and functional heterogeneity, allow greater understanding and provide subsidies for appropriate experimental design choices to test scientific hypotheses.

### 3.3. Arteriogenesis

Arteriogenesis refers to the formation of collateral arterioles, allowing increased blood flow to tissues and being a crucial compensation mechanism to restore tissue perfusion where the main vascular pathway has been obstructed [131]. In adults, arteriogenesis can occur in response to various stimuli, including changes in haemodynamic forces, such as shear stress, and in the metabolic demands of tissues, as in hypoxic conditions [132]. For this reason, postnatal arteriogenesis plays an essential role in restoring blood supply in several pathological situations, in which the metabolic demand is greater than the amount of blood perfusing the tissue [132, 133].

The arteriogenic process may occur by two distinct mechanisms,* de novo* formation, or remodelling of preexisting collateral arterioles. The first one involves the formation and expansion of new vessels from a preexisting arteriolar network, localised near to an occluded artery, reconnecting the distal arterial segment [26]. The second one involves the remodelling of preexisting vessels, promoting gradual enlargement of arterioles until they are able to increase blood flow and restore local perfusion [134].

During embryonic development,* de novo* arteriogenesis consists in the differentiation and maturation of the primary vascular plexus, induced by the increase in blood pressure. The consequent increase in shear stress in the new capillary network stimulates local recruitment of smooth muscle cells and proliferation of endothelial cells, leading to the formation of mature arteries [135–137]. There is evidence that arteriogenesis by* de novo* formation can also happen in the adult; however, this issue remains controversial. Some authors believe that the newly detected collaterals could be native collaterals not detected before remodelling or even preexistent capillaries that undergo arterialisation [138–140]. Nevertheless, the presence of new collateral arterioles was detected after acute arterial occlusion in murine brain [141] and heart [26], as well as after apical resection in neonatal murine heart [142]. Of note, these new collateral formations occurred in areas with apparent absence of preexisting connections. In addition, one cannot rule out the possibility of remodelling occurrence not only in preexisting collaterals, but also in the presumed* de novo* formed arteriole to increase their calibre, making this discussion even more complex and difficult to address* in vivo*.

Considering the difficulty to distinguish new arteriole formation from remodelling of preexistent ones, the mechanisms involved in* de novo* arteriogenesis remain poorly understood. The augment of shear stress in the arterial tree along with tissue hypoxia generated by a major artery occlusion is believed to be the main trigger for the beginning of the* de novo *process [26, 141]. Moreover, it was recently demonstrated that neo-collateral formation is dependent on local CCL-2/MCP-1 release and the recruitment of circulating CCR2^+^ cells to the ischaemic area in the murine heart. The recruitment of CX3CR1^+^ M2-like macrophages to the border zone of newly formed collaterals also appears to be associated with the growth of new arterioles [26].

While evidence about* de novo* arteriogenesis in adults is scarce, the dominant form of postnatal arteriogenesis appears to be largely dependent on the remodelling of preexistent collateral vessels [143]. The postnatal collateral remodelling may be divided into three phases: initiation, growth, and vessel maturation. When a major vessel occlusion promotes the drop-in of perfusion pressure at a distal point, this reduction leads to an increased flow through preexisting collateral arteries. The resultant increased wall shear stress is the main trigger for the initiation phase of arteriogenesis [144].

Shear stress activates the endothelium of collateral vessels. Activated endothelial cells, by their turn, stimulate the recruitment of local and bone marrow inflammatory cells, through the expression of chemokines as tumour necrosis factor (TNF), VEGF, and CCL-2 and adhesion molecules such as selectins, intercellular adhesion molecule 1 (iCAM1), and vascular adhesion molecule 1 (vCAM1) [133, 145]. The early recruited inflammatory cells are neutrophils, which help to degrade extracellular matrix, enabling vessels to expand [146]. The released CCL-2 chemokine, in turn, promotes the recruitment of circulating monocytes expressing the receptor CCR2 to the affected region [147, 148].

During the growth phase, the macrophages play an essential paracrine role, recruiting other bone marrow cells as well as modulating smooth muscle and endothelial cell proliferation. Macrophages orchestrate these actions by secretion of chemokines and growth factor as stromal derived factor 1 (SDF-1), PDGF-B, TNF, VEGF, and FGF [149]. In addition, macrophages modulate matrix remodelling by aiding on basement membrane degradation, through MMP-2 and MMP-9 secretion [150, 151].

The inflammatory signalling cascades induce the differentiation of smooth muscle cells from a contractile phenotype to a synthetic phenotype. This differentiation is characterized by the downregulation of actin, myosin, desmin and calponin expression, and upregulation of fibronectin [152]. The replacement of contractile material for endoplasmic reticulum and free ribosomes is also observed in smooth muscle cells [133, 153]. SMC with this phenotype are directed to migration, proliferation, and production of a fibronectin-based transitional matrix [133], essential for the remodelling process. The proliferation of smooth muscle and endothelial cells leads to collateral vessel luminal expansion and tortuous elongation. As luminal diameter increases, distal perfusion is restored and blood flow resistance decreases, as well as shear stress.

Finally, the collateral vessel passes through the maturation phase. With the decline on shear stress, the endothelium function of collateral vessel normalises and the inflammatory response decreases, initiating inflammation resolution and reduction of cell proliferation, as well as the return of SMC to a contractile phenotype [133]. At this point, the arterioles with lower resistance provide most of the blood flow and continue to remodel outward, stabilising and maturing into dominant collateral. At the same time, small collateral vessels that may not maintain sufficient hemodynamic stimulation undergo neointimal hyperplasia and eventual regression [154, 155].

Important signalling pathways associated with neovascularisation processes will be better discussed later in this manuscript. However, it is interesting to point out how some of these pathways can mediate distinct actions during arteriogenesis in different organs and tissues. The activation of the PI3K/AKT/eNOS pathway on EC, for example, plays an important role for adaptative collateral vessel remodelling in the ischaemic heart and hindlimb skeletal muscle. The resultant NO production and release by EC exert an important vasodilatation effect, increasing the flow through collateral vessels and, thereby, possibly augmenting flow-induced remodelling [139]. In addition, this axis activation is essential for sustained interactions of the endothelium with pericytes and vascular SMC, as well as maintenance of vascular stability [156, 157]. In the heart and retina, in turn, endothelial AKT signalling activation is essential to jagged/NOTCH signalling activation, which is shown to be crucial for vascular SMC survival [157]. Jagged/NOTCH signalling activation also plays an essential role in skeletal muscle-induced arteriogenesis in ischaemic hindlimbs. The pathway activation after ligation to delta-like ligand (Dll) from arterioles EC leads to perivascular macrophage maturation and antiinflammatory polarisation, improving collateral remodelling [156].

On arterial occlusive diseases, such as peripheral artery disease, myocardial infarction, and ischaemic stroke, the restoration of blood flow to the affected tissue must be quick so its viability and function can be preserved. Collateral vessels can effectively bypass arterial obstructions and are more likely to induce effective reperfusion of tissues than proliferation of capillary network [158]. However, the number of arteriolar collaterals was shown to vary widely among organs as heart, brain, and lower extremities, and even among individuals. In fact, clinical outcome in patients with arterial occlusive diseases appears to be directly correlated to the number of preexistent collaterals in the affected area and their capacity of remodelling [159, 160]. It was shown that the arteriole network remodelling process also can negatively affect tissues extensively damaged by hypoxia. In ischaemic hindlimbs, the formation of new arterioles with an aberrantly smooth muscle cells cover was observed, presenting increased interprocess spacing and haphazard actin microfilament bundles [161], which affected the restoration of blood perfusion effectiveness.

Interestingly, animal model studies have shown that even the speed of the arteriogenesis process may vary among different organs and tissues. For example, it was observed that the maximum remodelling of collaterals in murine ischaemic brain occurs up to 3 days after arterial occlusion [141], significantly faster than in the ischaemic heart (3 to 7 days) [26] and skeletal muscle (3 to 4 weeks) [162, 163]. The reason for this difference still needs to be elucidated, but one hypothesis suggested is that specific tissue characteristics in the location adjacent to the collateral vessels can affect the time required for reorganisation of the surrounding matrix and cells before outward remodelling can proceed [141].

In conclusion, arteriogenesis is a complex multifactorial process, stimulated by changes in the haemodynamic forces and in the metabolic demands, and is essential to restore the adequate blood supply to organs and tissues after acute or chronic major artery occlusion. Understanding and clarifying the heterogeneity of mechanisms involved in this neovascularisation process are crucial to develop effective clinical approaches for many arterial obstructive diseases.

### 3.4. Lymphangiogenesis

The lymphatic system is known for its primary function of draining fluids in the interstitial space, redirecting them to blood vessels. It is a drainage system, from and to which immune cells responsible for the defence of the organism may emerge and go [164]. In addition to this function, it is active in other contexts, namely, in the regulation of blood pressure, in the differentiation and modulation of immune and inflammatory cells, in lipid metabolism, and in atherosclerosis and metastasis. In turn, the physiological role of lymphatic vasculature is conditioned by other pathological events, such as obesity, chronic inflammation, and autoimmune diseases [165].

Lymphatic vessels present a morphological heterogeneity, being, therefore, subdivided into capillaries and collectors. The capillaries are responsible for the absorption of the extravasated fluid, whereas the collectors are transporters. With regard to the cellular morphology, capillaries are formed by button-type joints and collectors are made of the zipper types. In addition, lymphatic endothelial cells from capillaries are surrounded by dendritic cells, recruited by chemokines such as CCL-21, and expressing high levels of the lymphatic vessel endothelial receptor 1 (LYVE-1), in opposition to lymphatic EC from collectors [164].

Lymphatic capillaries are formed after the establishment of blood vasculature in a process in which venous endothelial cells undergo lymphatic speciation by presenting markers of the lymphatic vasculature such as LYVE-1, prospero homeobox protein 1 (PROX-1), and VEGFR-3 [166]. The mechanism by which new lymphatic vessels are formed is called lymphangiogenesis and, in the embryonic period, some veins present a lymphatic capacity when they present endothelial cells that express PROX-1, SOX-18, and LYVE-3. At a later stage, they begin to express VEGFR-3 and, following a gradient concentration of VEGF-C, they migrate, proliferate, and form a primitive lymphatic sac, expressing new lymph markers and originating a lymphatic plexus where maturation and remodelling occur. Smooth muscle cells are recruited and form leaflets that allow the lymphatic vessel to become functional. In addition, novel lymphatic markers are then expressed such as podoplanin, ephrin B2 (EFNB2), forkhead box protein C2 (FOXC2), angiopoietin 2 (ANGPT2), integrins, neuropilin 2 (NRP2), and emilin [167].

The expansion of this vascular network occurs mainly by lymphangiogenesis, although in recent years the process of lymphovasculogenesis has been referred to as a possible process that occurs after the embryonic phase. During lymphangiogenesis, LEC from preexisting vessels proliferate and migrate, process which involves VEGF-C, collagen and calcium binding EGF domains 1 (CCBE1), and disintegrin and metalloproteinase with thrombospondin motif 3 (ADAMTS-3) that stimulate VEGFR-3 and NRP2 receptors in the lymphatic endothelial cell. In lymphovasculogenesis, the haemogenic endothelium produces haematopoietic stem cells (HSC) that will serve the new lymphatic vascular formation, similar to what occurs in the process of vasculogenesis [168].

The main difference between lymphovasculogenesis and lymphangiogenesis is the origin of the cells that give rise to the vasculature. In the first context, the cells come from preexisting vessels, and in the second they are derived from the haemogenic endothelium or, as more recently discussed, from circulating progenitor cells that were transdifferentiated into lymphangioblasts, revealing heterogeneity in the cells that originated from lymphatic vessels [164, 169]. Although studies from 2010 already addressed the existence of circulating lymphatic progenitor cells, only more recently these reports become more usual. Lee and colleagues [170] have demonstrated that bone marrow-derived cells expressing the protein podoplanin, characteristic of lymphatic vasculature, contributed to postnatal neovascularisation, indicating which progenitor cells could participate in lymphatic neoformation. These authors characterized these cells immunophenotypically and demonstrated that they express lymphatic cell markers such as LYVE-1 and VEGFR-3, besides podoplanin when cultured* in vitro*. Likewise, when these cells were tested* in vivo* in cell therapy models, they incorporated the neoformed lymphatic vasculature in corneal, ear, skin, and tumour models, and continued to express the markers of lymphatic cells. These findings open a promising field of study and provide new perspectives to the treatment of diseases that affect the lymphatic system. New approaches have been focused on determining markers, the origin, and niches for isolation of lymphatic precursor cells [21, 169], on generating pure lymphatic endothelial cells from pluripotent stem cells and studying their effects on wound repair [171], and on clarifying the mechanisms by which lymphatic endothelial precursor cells are mobilised in pathological and physiological contexts [172].

Lymphangiogenesis occurs physiologically in restricted situations, such as during the embryonic period for expansion of the lymphatic network of the embryo, during female reproductive cycle, and in mammary gland genesis [173]. In fact, lymphangiogenesis is more seen occurring in pathological conditions. Lymphatic vascular dysfunction can cause oedema formation, due to failed draining of the interstitial contents, or may contribute to the formation of new lymphatics that are associated with metastatic dissemination to distant lymph nodes and organs, and rejection of grafts. Lymphangiogenesis is also seen during obesity, since excess lymph in the interstitium leads to the production of proinflammatory cytokines and hypertrophy of adipocytes. Lymphoedema is classically observed in the infectious disease filariasis, but also in some cardiovascular and genetic conditions [174].

Similar to angiogenesis, this process involves three general steps, among which are the activation and migration of lymphatic endothelial cells, their proliferation, and lumen formation. Lymphangiogenic factors such as PDGF, tumour necrosis factor *α* (TNF), VEGF, and MMP bind receptors on the surface of lymphatic endothelial cells that become activated and stimulate the sprouting process. In this case, the profile selection into either tip or stalk cells also occurs, although the factors and pathways coordinating it are not well defined as for angiogenesis. Tip cells begin to migrate and emit filopodia, while stalk cells continue proliferating and allowing vessel elongation. Lumen formation occurs by intracellular vacuolisation and, finally, there is remodelling and maturation of the vessel [175, 176]. VEGF is also the main factor to stimulate lymphatic sprouting. Lymphatic EC migrate toward VEGF gradient, mainly in a VEGFR-3-dependent manner, and EFNB2 promotes lymphatic EC migration by regulating cell internalisation of VEGFR-3. Differently from sprouting angiogenesis, the NOTCH pathway is not active in lymphangiogenesis [177].

Lymphangiogenesis during inflammatory conditions is also mediated by VEGF. Inflammatory macrophages secrete large amounts of VEGF and stimulate local lymphatic sprouting, which aids in the supply of inflammatory cells and drainage of exudate. In this process, VEGF-C/VEGFR-3 and VEGF-A/VEGFR-2 signalling pathways are known to have a significant role, although other pathways are involved. It has also been argued that inflammatory macrophages could become incorporated into lymphatic vessels and transdifferentiate into lymphatic endothelial cells, but this hypothesis is not fully accepted by the scientific community [178]. Some studies, however, have demonstrated the important role of macrophages in the formation of lymphatic vessels. They are named lymphangiogenic macrophages and, during inflammatory processes, they show direct and indirect effects on lymphangiogenesis. Direct effects were related to the production of prolymphangiogenic factors, and the indirect ones were attributed to their contribution to the mobilising of more macrophages from bone marrow and the amplifying of the immune response, further stimulating lymphangiogenesis. In addition, the activation of Toll-like receptors (TLR) during inflammatory contexts leads to increased production of VEGF-C and -D by macrophages, inducing the proliferation of lymphatic endothelial cells [179, 180].

In a tumoural context, lymphangiogenesis is associated with tumour malignancy. Tumour lymphatic vessels as well as blood vessels are compromised. Tumour cells and tumour stroma release lymphangiogenic factors that increase the sprouting of lymphatic vessels, which are also essential for tumour progression. In addition, tumour cells release VEGF-C and express both VEGFR-3 and CCBE-1, stimulating lymphangiogenesis. Tumour-associated macrophages can secrete lymphangiogenic factors as well as incorporation into the new lymphatic vasculature, contributing to lymphangiogenesis and dissemination of micrometastases and metastases to distant lymph nodes and organs [181, 182].

The interaction of tumour cells with lymphatic cells can be promoted by interstitial fluid (resulting from lymphatic drainage) via autologous chemotaxis involving the chemokine CCL-21 and its receptor, CCR-7, expressed by tumour cells. Therefore, the expression of CCL-21 in lymphatic EC can promote the entry of tumourigenic cells into lymphatic vessels via CCR-7. The production of lymphangiogenic factors such as VEGF-C and -D stimulates the formation and enlargement of lymphatics in the vicinity of the tumour, increasing the surface area for interaction between the cells. VEGF-C also promotes the invasiveness of tumour cells in an autocrine manner and upregulates the production of CCL-21 by lymphatic vessels [183]. In view of this knowledge, as well as in angiogenesis, some therapeutic strategies involving the use of antilymphangiogenic agents and blocking of VEGF signalling have been proposed and studied to successfully inhibit tumour growth. Anti-VEGF and anti-VEGF-C monoclonal antibodies, as well as antibodies against specific parts of the VEGFR-2 receptor, were produced, yet there are no approved drugs able to only block lymphangiogenesis, which could benefit some pathologies that do not necessarily require the blocking of angiogenic processes [183, 184].

## 4. Cellular Heterogeneity and Blood-Organ Barriers

Different processes of neovascularisation occur in response to a range of stimuli, factors, and mediators. Furthermore, different organs and tissues exhibit distinct biological roles, and their metabolic demands and physiological processes can be maintained by modulating cellular and molecular factors associated with the neovascularisation processes above-mentioned. Additionally, the phenotype of the cells that constitute the blood-organ barriers, especially endothelial and mural cells, seems to be important during the establishment of new vessels and is also related to the heterogeneity associated with the microvascular system. In this section, we will address the heterogeneity of factors related to endothelial and mural cells (pericytes and smooth muscle cells) in the functional and neovascularisation contexts related to specific blood-organ barriers.

### 4.1. Endothelial Cells

EC are found in every vascular bed, including lymphatics, and their cellular and functional heterogeneity is recognised for decades. They produce autocrine and paracrine molecules, regulating cell adhesion, proliferation, vessel permeability, and the migration of blood cells throughout the endothelium. They also share some common features such as their morphology—small cells that possess few mitochondria and both granular and agranular endoplasmic reticulum, whose diameter across their thin cytoplasmic section is no longer than 0.2 *μ*m and which are 5-10 *μ*m wide [80].

**Figure 3:**
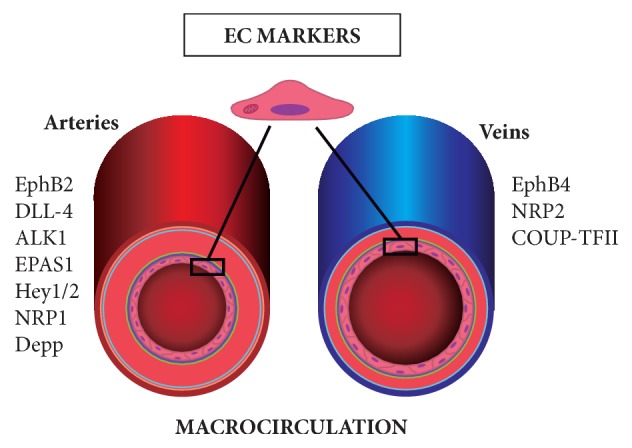
**Molecular profiles of endothelial cells in the macrocirculation. **Markers preferentially expressed by EC from arteries and veins. EphB2: ephrin type-B receptor 2. EphB4: ephrin type-B receptor 4. DLL-4: delta-like 4. ALK1: activin receptor-like kinase 1. EPAS1: endothelial PAS domain-containing protein 1. Hey1/2: hairy/enhancer-of-split related to YRPW motif protein 1/2. NRP1: neuropilin 1. NRP2: neuropilin 2. Depp: decidual protein induced by progesterone. COUP-TFII: COUP transcription factor 2.

The phenotypic differences can be observed in different vascular beds and in different segments of the same vessel. EC from arterial and venous portions, for example, express unique molecular signatures. On the one hand, EphrinB2 (EphB2), delta-like 4 (DLL-4), activin-receptor-like kinase 1 (ALK1), endothelial PAS domain protein 1 (EPAS1), Hey 1 and Hey 2, neuropilin (NRP1), and decidual protein induced by progesterone (Depp) are preferentially expressed in arterial EC [185–189]. On the other hand, venous EC specific markers include EphB4, NRP2, and chicken ovalbumin upstream promoter transcription factor 2 (COUP-TFII) [190–192] (Figure 3).

**Figure 4:**
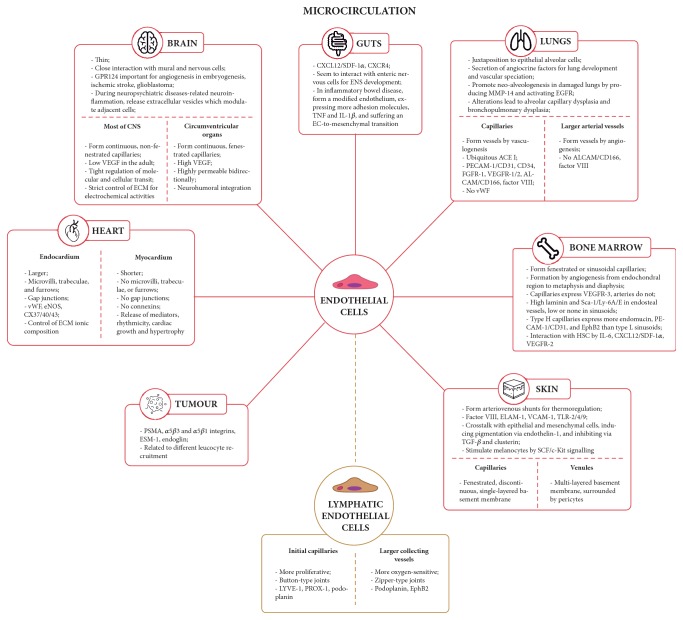
**Multifocal heterogeneity of endothelial cells* per* organ-blood barrier. **Morphofunctional, organisational, and molecular specificities of EC forming the microvasculature of different vascular beds, from organotypic, tissue-related, and vessel-specific perspectives in physiological and pathological contexts. vWF: von Willebrand factor. eNOS: endothelial nitric oxide synthase. CX37/40//43: connexin 37/40/43. ECM: extracellular matrix. GPR124: probable G-protein coupled receptor 124. VEGF: vascular endothelial growth factor. CXCL12: C-X-C motif chemokine ligand 12. SDF-1*α*: stromal cell-derived factor 1 alpha. CXCR4: C-X-C chemokine receptor type 4. ENS: enteric nervous system. TNF: tumour necrosis factor. IL-1*β*: interleukin 1 beta. EC: endothelial cell. MMP-14: matrix metalloproteinase 14. EGFR: endothelial growth factor receptor. ACE I: angiotensin-converting enzyme I. PECAM-1: platelet endothelial cell adhesion molecule 1. CD31: cluster of differentiation 31. CD34: cluster of differentiation 34. FGFR-1: fibroblast growth factor 1. VEGFR-1/2: vascular endothelial growth factor receptor 1/2. ALCAM: activated leukocyte cell adhesion molecule. CD166: cluster of differentiation 166. VEGFR-3: vascular endothelial growth factor receptor 3. Sca-1: stem cells antigen-1. Ly-6A/E: lymphocyte antigen 6A/E. EphB2: ephrin type-B receptor 2. HSC: haematopoietic stem cell. IL-6: interleukin 6. ELAM-1: endothelial-leukocyte adhesion molecule 1. VCAM-1: vascular cell adhesion protein 1. TLR-2/4/9: Toll-like receptor 2/4/9. TGF-*β*: transforming growth factor beta. SCF: stem cell factor. c-Kit: tyrosine-protein kinase kit. LYVE-1: lymphatic vessel endothelial hyaluronan receptor 1. PROX-1: prospero homeobox protein 1. PSMA: prostate-specific membrane antigen. ESM-1: endothelial cell-specific molecule 1. Organ icons obtained from the Noun Project website (https://thenounproject.com): Brain by David; Heart by PJ Witt; Lungs by Ayub Irawan; skin by Hermine Blanquart; Guts (named intestine) by Anthony Bossard; Bone (named Pacifier) by corpus delicti; Tumour (named disease) by Viral faisalovers from the Noun Project.

Phenotypic differences are also observed in the microvascular context, reflecting a range of compartments in the same organ or tissue, as it will be highlighted throughout this section. In addition, it is worth noting that these differences may be crucial in determining the type of neovascularisation in a particular vascular bed or tissue under physiological and/or pathological conditions. The heterogeneity existing in different blood-organ barriers will also be addressed regarding EC differences and how it is related to the establishment of an organ-specific function (Figure 4).

#### 4.1.1. Blood-Heart Barrier (BHB)

Organs like the heart, for example, present several endothelial compartments and their EC present differences related to their developmental origin, structure, and functions. The EC-rich cardiac compartments include the endocardial endothelium (EE) and the vascular endothelium of myocardial capillaries (VEMC). The EE features, such as larger size, presence of microvilli, and abundant gap junctions, are consistent with its function in controlling myocardial extracellular ionic composition and as a sensor of blood flow, while myocardial EC functions are more related to autocrine or paracrine mediator release, rhythmicity, and cardiac development and growth, for example, during hypertrophy [193].

The normal development, formation, and maturation of heart are dependent on the transformation of the ventricular wall from a trabecular network into a dense myocardium during gestation. In this sense, endocardium plays an important role in the transition, given its coronary progenitor tissue capacity. Failure in this process could trigger defective coronary vascularisation and consequently congenital cardiac diseases [194]. Recently, Rhee and colleagues [195] have shown that a normal cardiac development is dependent not only on the neovascularisation capacity, but also on the paracrine muscle growth support given by active EC. They observed that the deletion of the Ino80 complex, a conserved multisubunit chromatin remodeller, causes intermediate phenotypes in EC from EE and is associated with impaired coronary angiogenesis and heart wall compaction in mice. These data corroborate previous observations that the different EC from the heart endothelium compartments may secrete different angiocrine factors that modulate processes associated with the homeostasis in the heart [196, 197].

#### 4.1.2. Blood-Brain Barrier (BBB)

Intraorgan EC heterogeneity also contributes to regional specific functions in the Central Nervous System (CNS). Most regions of the CNS have continuous nonfenestrated capillaries with BBB properties, i.e., which tightly regulate the movement of molecules, ions, and cells between the blood and the CNS, as part of the neurovascular unit. In contrast, specific regions such as nuclei adjacent to the third and fourth ventricles, pineal gland, and median eminence show highly permeable continuous fenestrated vessels [198].

Microvascular EC from CNS are thin cells, roughly 39% less thick than skeletal muscle EC, that coordinate junctional, transport, and metabolic aspects of the BBB by means of close interaction with mural, immune, glial, and neural cells for maintaining the brain functions [199, 200]. The vascular system in the CNS originates by angiogenesis from the perineural vascular plexus sprouting into the embryonic neuroectoderm. While the general angiogenic pathways, such as VEGF/VEGFR, Delta/NOTCH, angiopoietin/Tie, and ephrin/Eph, are required for vascular development in the CNS, the EC expression of the G-protein-coupled receptor 124 (GPR124), which is a coactivator of the Wnt signalling, plays a central role in that process, thus representing an important contributor to the BBB establishment [201–203]. In addition, GPR124 is also related to the BBB integrity during pathological conditions, such as ischaemic stroke and glioblastoma, in adult mice [204].

Of note, although there is a close correlation between the expression pattern of VEGF and brain angiogenesis during development, VEGF levels decrease during the late embryonic development when capillaries become impermeable to form the BBB in rodents, reaching very low levels in the adult brain, consistent with a undesirable permeability effect of VEGF. In contrast, fenestrated capillaries show a high constitutive expression of VEGF [205].

Brain vasculature dysfunctions have been documented in vascular malformations, Alzheimer, stroke, epilepsy, and traumatic brain injuries [206]. Neuroinflammation is usually associated with these neurological diseases by promoting increased permeability of the BBB. During neuroinflammation, EC from BBB, for example, release extracellular vesicle (EV) subsets, such as microvesicles (MV) and exosomes (EXO), which are able to induce phenotypic changes and impacting physiology of adjacent cells, such as astrocytes, pericytes, and microglia [207, 208], suggesting an important role of EC for brain, beyond their barrier function, in the homeostasis and pathological conditions in CNS.

#### 4.1.3. Gut-Blood Barrier (GBB)

The gut-blood barrier controls the biological compounds which enter in the systemic circulation, so the integrity and permeability of their capillaries should be strictly controlled avoiding the wide pass of gut-derived molecules, but allowing entry of nutrients across GBB [209, 210]. Epithelial and gut EC make coordinating responses which avoid activation of local immune responses and microbial dissemination, participating on gut tissue homeostasis [211]. As to the characteristics of the GBB, Spadoni and colleagues [210] found similarities regarding the expression of junctional complexes formed by tight and adherent junctions in EC from brain and intestine, a predictable finding since both systems behave as selective barriers.

A diversity of factors is involved in gastrointestinal tract vascularisation. An important pathway is the CXCL-12/CXCR-4 axis. EC from mice lacking CXCR-4 or CXCL-12/SDF-1*α* are not able to form large vessels and show impaired microvasculature in the gastrointestinal tract [212]. Controversial findings related to influence of gut EC in the enteric nervous system (ENS) development are found. Fu and colleagues [213] hypothesized that gut EC play a critical role in promoting and directing the development of the ENS. In contrast, Delalande and colleagues [214] showed that blood vessels are not required to ENS development in mice. However, as the ENS is compromised during impaired neovascularisation, it is possible to credit the importance of enteric nervous cells (ENC) and EC interactions prior to the establishment of the neurovascular units seen in the gut later in development.

The proper functioning of the GBB may be altered in several diseases, such as inflammatory bowel disease (IBD), and is somehow correlated with pathological neovascularisation. During the progress of IBD, angiogenesis occurs in response to inflammatory growth factors, cytokines, and chemokines released by inflammatory cells in the gastrointestinal system. The EC are responsible for coordinating vascular supply and immune cell emigration, for example. Of note, the newly formed endothelium is distinct to the normal vessel and their EC express higher levels of adhesion molecules and release proinflammatory cytokines such as TNF and IL-1*β*, contributing to IBD progression and severity [215]. In addition, abnormalities in the GBB caused by infectious agents as* Salmonella sp. *result in endothelial gene expression profile alteration and EC-to-mesenchymal transition [210].

Although the hepatic endothelium is quite close and associated with the GBB, a morphological and functional heterogeneity is also seen in that organ as a result of a range of EC phenotypes. EC from portal venules are spindle-shaped and nonfenestrated and possess short microvilli. In contrast, EC from terminal portal venule and hepatic sinusoid capillaries are smooth and large and contain many actin fibres [216, 217].These differences correlate to the specific biological functions of those hepatic segments. Another important characteristic of the hepatic sinusoidal vasculature refers to the EC capacity in mediating organogenesis and liver regeneration by stimulating the production of the hepatocyte growth factor (HGF) [218] together with the release of angiocrine factors to promote hepatocyte proliferation [219, 220].

#### 4.1.4. Alveolar-Blood Barrier (ABB)

The alveolar-blood barrier is composed of a thin layer of EC in juxtaposition to the surface of alveolar epithelial cells. The development and vascular speciation of the lungs occurs in response to angiocrine factors secreted by primitive capillaries. Postnatally, a high vascular refinement, remodelling and maturation occurs [221–223]. Alterations in these processes may lead to the development of abnormalities such as alveolar capillary dysplasia and bronchopulmonary dysplasia.

Pulmonary capillary EC are identified by their expression of PECAM-1/CD31, CD34, FGF receptor 1 (FGFR-1), VEGFR-1, and VEGFR-2 [224]. Moreover, they express the angiotensin I-converting enzyme (ACE I), compared with about 10% of systemic capillary EC, which, in turn, express PECAM-1/CD31 and CD34, but not vWF, similar to endocardial EC. An intrinsic heterogeneity of EC is also observed in pulmonary vasculature, since activated leukocyte cell adhesion molecule (ALCAM/CD166) and the factor VIII are expressed in rat lung capillary endothelium, but not in larger pulmonary vessels [80, 225, 226].

Lung EC not only act as a selective and protective barrier, but also release angiocrine signals which may act in the pulmonary tissue homeostasis and during pathological conditions, by inducing a neo-alveologenesis in damaged lungs. Pulmonary capillary EC, for example, produce MMP-14 in response to a lung injury which in turn activates the EGF receptor (EGFR) that promotes proliferation of alveolar epithelial cells [227–229].

#### 4.1.5. Bone Marrow-Blood Barrier (BMBB)

The bone marrow-blood barrier main functions are related to the cell traffic control between the extravascular and intravascular marrow compartments [230, 231]. Structurally, the bone microvasculature is basically formed by fenestrated or sinusoidal capillaries whose EC express the vascular endothelial growth factor receptor-3 (VEGFR-3), while bone arterial endothelium does not [232]. The vascularisation process in bone formation occurs by angiogenesis from the embryonic endochondral region, and signals from plate chondrocytes at the ends of the developing long bone are responsible for the formation of the metaphyseal and diaphyseal capillary networks [233, 234]. Classically, the vascular network in bone is identified by a low laminin and/or no Sca-1 (Ly-6 A/E) expression in sinusoids, in contrast to the high expression of those markers in endosteal vessels [235]. Microvessel subtypes, called type H capillaries, that receive the bone arteriolar blood flow are found in growing regions of bone and express higher levels of Endomucin (EMCN) and PECAM-1/CD31 compared to the type L sinusoidal localised in the diaphysis that receives the type H blood flow. Of note, type H capillaries also express ephrin B2 (EphB2) and are believed to generate arteriolar blood vessels. From type L capillaries, blood is, then, drained to the central vein [236, 237].

In the light of the present discussion, the cell traffic is particularly important as cell source during vasculogenesis, the angioblast/endothelial progenitor cell-driven neovascularisation process that occurs mainly during the embryonic development, but is also assumed to contribute to new blood vessel formation from circulating stem/progenitor cells in the postnatal period. In addition, the traffic of haematopoietic lineages, decidedly important for blood cell reposition and during immune responses and immunosurveillance, is also relevant to neovascularisation processes [238].

Given the diversity of cell sources and functions, different metabolic environments are therefore expected to be found in the bone marrow. This richness of environments is supported by a heterogeneous, unique bone vasculature with specialised functions. Indeed, multiple vascular niches in BM have been discovered unveiling its complex cell heterogeneity, which reflects the intricacy of interactions and coordinated functions of the BM [239].

Interactions between BMB EC and haematopoietic stem cells (HSCs), for example, are important to haematopoiesis, stem cell mobilisation, and homing [239–241]. In fact, molecular signals in BMB EC, such as IL-6, CXCL-12, VEGFR-2 signalling, E-selectin expression, and stem cell factor (SCF), are known to modulate HSC homeostasis [242–245]. The knowledge about the expression of molecules and angiocrine factors in different niches of BM is important to understand the fine-tuning on cell production and traffic regulation in a range of physiological and pathological processes; however, this is still a subject that needs more refinement.

#### 4.1.6. Skin-Blood Barrier (SBB)

In the skin, dermis and hypodermis are richly supplied by blood and lymphatic vessels, mainly involved in thermoregulation, wound healing, and immune reactions. They can be subdivided into nutritional and thermoregulatory vessels, with the nutritional capillaries carrying less than 15% of the total of cutaneous blood flow. Particularly, the skin microvascular network is composed by capillaries, venules, arterioles, and some specialised arteriovenous shunts which act as sphincters, allowing the capillary circulation to be short-circuited when they are open. Skin capillaries are fenestrated, discontinuous, and surrounded by a simple basement membrane, whereas postcapillary venules have a multilayered basement membrane surrounded by pericytes, a type of mural cell [246]. These features allow the capillaries skin EC to form an interface between intravascular and extravascular compartment by exhibiting a selective barrier for diffusion of macromolecules and cells across the, so-called, skin-blood barrier (SBB) [247].

In health conditions, microvascular SBB EC express factor VIII, endothelial cell leukocyte adhesion molecule 1 (ELAM-1), and vascular cell adhesion molecule 1 (VCAM-1). In addition, SBB act as a secondary barrier against pathological agents and play part of the innate immune response during skin injury by expressing a range of receptors of the Toll-like family, such as TLR2, TLR4, and TLR9, which are involved in pathogen recognition [248].

Another particular function associated with EC heterogeneity in skin is related to the skin hyperpigmentation. A possible crosstalk between microvascular EC and epithelial and mesenchymal cells in the cutaneous compartment has been suggested. In physiological conditions, Endothelin-1 promotes skin pigmentation while transforming growth factor (TGF-*β*) and Clusterin inhibit the pigmentation. All these factors are produced by dermal EC, indicating a role of them in modulating pigmentation process [249, 250]. In accordance, interactions between dermal EC and melanocytes have also been observed. Kim and colleagues [251] showed that UV-increased melanocyte pigmentation of* ex vivo *human skin is dependent on SCF/c-Kit signalling, suggesting that melanogenic factors are released by EC during chronic sun exposure. In summary, SBB EC present unique characteristics that permit them to act as modulators of melanocyte and epithelial cell homeostasis and may play an important role in skin protection.

#### 4.1.7. Lymphatic Endothelial Cells (LEC)

Lymphatic endothelial cells also exhibit heterogeneity and cellular plasticity [252]. Few studies, however, have explored the tissue-specific heterogeneity in LEC. They demonstrate a significant difference in gene expression from initial/capillary (micro) to collecting (macro) lymphatic vessels. Capillary LEC, for example, express LYVE-1 and podoplanin, whereas collecting lymphatic vessel EC express podoplanin and EphB2 [253, 254].

The biological responses of LEC reflect this heterogeneity and are known to be condition-dependent. For example, capillary LEC are more proliferative and less oxygen-sensitive compared to LEC from collecting lymphatics. In addition, organ-specific heterogeneity of LEC is observed in isolated cells from lymph node, spleen, thymus, palatine tonsil, and iliac lymphatic vessel in relation to patterns of vascular markers expression [255, 256].

#### 4.1.8. Pathological Conditions-Related EC Heterogeneity

The pathological context can also drive EC heterogeneity. EC from tumour present different endothelial markers compared to EC in normal tissues and react differently to antibodies. In this sense, prostate-specific membrane antigen (PSMA), for example, was shown to be expressed in tumour endothelium, but not in normal vascular endothelium. Other markers increasing in tumoural EC include *α*5*β*3 and *α*5*β*1 integrins, endothelial-specific molecule 1 (ESM-1), and endoglin [257]. EC heterogeneity also influences the progression of inflammatory processes, revealing distinct patterns of inflammation-induced changes in protein expression and leukocyte recruitment [258].

Overall, the current knowledge discloses a high plasticity in EC phenotype and functions that represent opportunities for targeting molecules and markers of health and disease. More studies emphasizing these differences are important to elucidate the origin, heterogeneity mechanisms, and paracrine factors release by them in different vascular beds and health conditions.

### 4.2. Mural Cells

**Figure 5:**
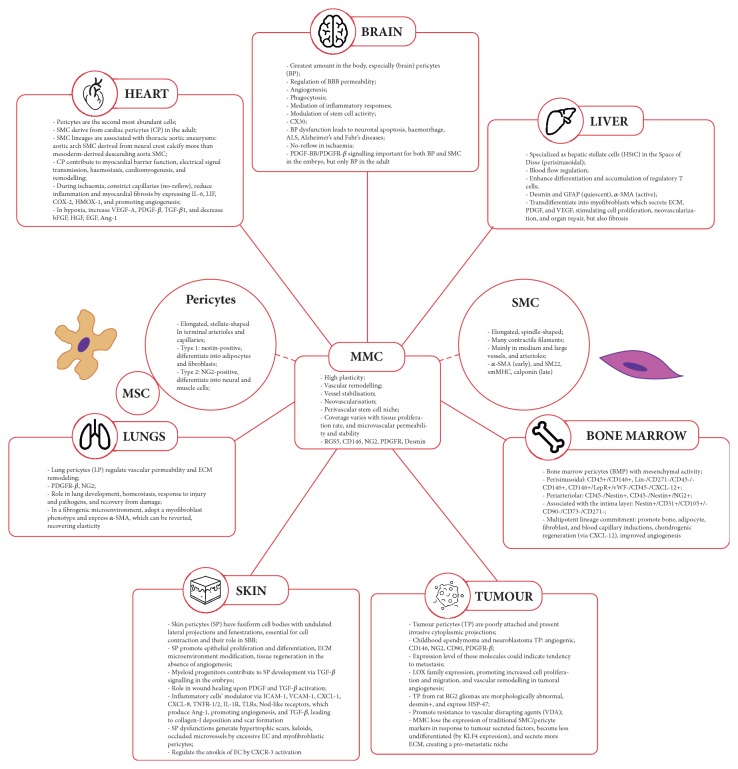
**Multifocal heterogeneity of mural cells* per* organ-blood barrier. **Morphofunctional, organisational, and molecular specificities of pericytes and smooth muscle cells in different organs, also in physiological and pathological contexts. SMC: smooth muscle cell. CP: cardiac pericyte. IL-6: interleukin 6. LIF: leukaemia inhibitory factor. COX-2: cyclooxygenase 2. HMOX-1: heme oxygenase 1. VEGF-A: vascular endothelial growth factor A. PDGF-*β*/PDGFR-*β*: platelet-derived growth factor receptor beta. TGF-*β*1: transforming growth factor beta 1. bFGF: basic fibroblast growth factor. HGF: hepatocyte growth factor. EGF: endothelial growth factor. Ang-1: angiopoietin 1. BP: brain pericyte. BBB: brain-blood barrier. CX30: connexin 30. ALS: amyotrophic lateral sclerosis. PDGF-BB: platelet-derived growth factor BB. HStC: hepatic stellate cell. GFAP: glial fibrillary acidic protein. *α*-SMA: alpha-smooth muscle actin. ECM: extracellular matrix. PDGF: platelet-derived growth factor. VEGF: vascular endothelial growth factor. MSC: mesenchymal stem cell. NG-2: neuron-glial antigen-2. RGS5: regulator of G-protein signalling 5. CD146: cluster of differentiation 146. SM-22: smooth muscle protein 22. smMHC: smooth muscle myosin heavy chain. LP: lung pericyte. BMP: bone marrow pericyte. CD45: cluster of differentiation 45. Lin: lineage marker. CD271: cluster of differentiation 271. LepR: leptin receptor. CXCL-12: C-X-C motif chemokine ligand 12. CD31: cluster of differentiation 31. CD105: cluster of differentiation 105. CD90: cluster of differentiation 90. CD73: cluster of differentiation 73. SP: skin pericyte. SBB: skin-blood barrier. TGF-*β*: transforming growth factor beta. ICAM-1: intercellular adhesion molecule 1. VCAM-1: vascular cell adhesion protein 1. CXCL-1: C-X-C motif chemokine ligand 1. CXCL-8: C-X-C motif chemokine ligand 8. TNFR1/2: tumour necrosis factor 1/2. IL-1R: interleukin 1 receptor. TLRs: Toll-like receptors. Ang-1: angiopoietin 1. EC: endothelial cell. CXCR-3: C-X-C motif chemokine receptor 3. TP: tumour pericyte. LOX: lysyl oxidase. RG2: rat glioma 2. HSP-47: heat-shock protein 47. VDA: vascular disrupting agent. MMC: microvascular mural cell. KLF4: Kruppel-like factor 4. Organ icons obtained from the Noun Project website (https://thenounproject.com): brain by David; heart by PJ Witt; lungs by Ayub Irawan; skin by Hermine Blanquart; liver by Kamaluddin; bone (named pacifier) by corpus delicti; tumour (named disease) by Viral faisalovers from the Noun Project.

Microvascular mural cells (MMC) comprise pericytes and microvascular smooth muscle cells (SMC) and present high plasticity and organotypic functional roles in microvascular homeostasis and diseases. MMC are associated with neovascularisation and play important roles in vascular remodelling and vessel stabilisation. More recently, MMC have been considered to represent a perivascular stem cell niche, thereby providing a source for mesenchymal stem cells (MSC) [259, 260] (Figure 5).

Although MMC are a rising field of study, their phenotypic and organotypic heterogeneity, as well as their origin and specific markers, are still unclear [259]. Several sources have been identified,* in vivo*, such as MSC surrounding new blood vessels, neural crest cells, or cells in the secondary heart field which give rise to SMC in the aorta and pulmonary artery [259]. Although evidence indicates a common origin to MMC and MSC, they present different signalling pathways reflecting a range of different functions depending of tissue/organ of which they are part.

The existence of different embryogenic sources for distinct mural cell populations and vascular beds has been implicated in the genesis of diseases, especially in the cardiovascular system. A possible reason for this is based on the hypothesis that the distinct embryonic origin of MMC leads to differences in how the cells respond to disease mediators in different vascular regions. In thoracic aortic aneurysms, different SMC lineages were associated with the major risk for the disease, and aortic arch SMC derived from neural crest were shown to have a greater propensity to calcify compared to mesoderm-derived descending aorta SMC in a mouse model [261, 262]. In relation to the abundance MMC in the tissues, nervous system contains the greatest amount of MMC, whereas in skeletal muscle only one in 100 EC is covered by these cells, suggesting that the degree of MMC coverage is related to biological features of each tissue, such as proliferation rate and microvascular permeability and stability [259, 263].

Differences between MMC also are seen on vessel diameter and in subpopulations within the same vessel, besides morphological variances among these cells. SMC are found in medium, large blood vessels, and arterioles have contractility functions, regulating vascular resistance in the circulatory system, and present elongated, spindle-shaped cells with high concentration of contractile filaments. Besides, they have the ability to modulate their phenotype depending on microenvironment clues, assuming either a differentiated contractile or a synthetic proliferative phenotype [264]. Surface markers are specific for different SMC; alpha-smooth muscle actin (*α*-SMA), for example, is early expressed, while SM22 is higher expressed in differentiated SMC. Smooth muscle myosin heavy chain (smMHC) is found in mature contractile SMC, with calponin (a member of calmodulin family) being a later marker [265, 266].

Pericytes, in turn, are found in microvessels such as terminal arterioles and capillaries, with high densities in the brain, eyes, and kidneys [267]. They are stellate-shaped and are positioned adjacent to the endothelium and within the basement membrane [267]. The surface markers expression depends on tissue and developmental or angiogenic state of the vasculature. Among them, we may highlight desmin and *α*-SMA, regulator of G-protein signalling 5 (RGS-5), neuron-glial 2 (NG2), and PDGF receptor *β* (PDGFR-*β*) that are used to identify pericytes in tissues [268]. Two different subsets of pericytes, the so-called type-1 and type-2 pericytes, were identified by Birbrair and colleagues [269] using a double transgenic Nestin-GFP/NG2-DsRed mouse. In addition to their phenotypic differences, they also display distinct plasticity capacities, with type-1 being able to differentiate into adipocytes and fibroblasts and type-2 into neural and muscle cells. Nevertheless, despite their phenotypic heterogeneity, MMC may share some markers, such as RGS5, CD146, NG2, PDGFR-*β*, and desmin [270, 271].

#### 4.2.1. Heart

In the heart, MMC have been found in the functional units of the myocardium and in the tunica intima of coronary vessels, and pericytes constitute the second most abundant cells in the cardiac tissue [272]. Of note, recent findings support the idea that coronary SMC derive from pericytes in the adult heart [273]. Their central position (between endothelium and cardiomyocytes) makes the cardiac pericyte (CP) an important mediator in integrative functions. In fact, CP contribute to myocardial barrier function, electrical signal transmission, and haemostasis (by means of tissue factor and prothrombinase expression) as well as to cardiomyogenesis and remodelling [273–278]. During ischaemia, pericytes contraction contributes to no-reflow, causing constriction of capillaries, despite reopening of the affected artery [279]. In addition, they are also able to reduce inflammatory infiltration and myocardial fibrosis in infarct areas, by expressing immunoregulatory molecules, including IL-6, LIF, COX-2, and HMOX-1, and promoting angiogenesis on the heart tissue. In addition, hypoxia induces the expression of VEGF-A, PDGF-B, TGF-*β*1, and their receptors, while bFGF, HGF, EGF, and Ang-1 decreased the expression [280].

#### 4.2.2. Brain

The MMC in the brain are localised between the endothelium, neurons, and astrocytes and play roles such as regulation of BBB permeability, angiogenesis, phagocytosis, mediation of inflammatory responses, and modulation of stem cell activity. A morphological and molecular heterogeneity is observed in brain pericytes (BP) and a new marker has been described recently by Mazaré and colleagues [281]. Some brain regions show a subset of BP expressing connexin 30 (CX30), a gap junction protein, which was thought to be exclusively in brains astrocytes, but their function on the pericyte physiology remains unknown.

BP degeneration promotes the BBB breakdown leading to neurotoxic molecules accumulation resulting in neuronal dysfunction and apoptosis, cerebrovascular dysfunction, and amyotrophic lateral sclerosis. In addition, similar to ischaemic injuries on heart, BP can lead to a contractile rigor and obstruction of capillaries in brain [282–284]. Diverse signalling pathways are involved in the functions played by BP, and dysfunctions on these signalling also lead to pathologies development. The PDGF-B/ PDGFR-*β* signalling is important to brain microvasculature establishment, and dysfunctions on this pathway are associated with micro brain haemorrhages and Alzheimer's and Fahr's diseases, by either a reduction of PDGF production by EC or less expression of PDGF receptors by BP [283–285]. A recent study shows that PDGFR-*β*^F7/F7^ mice, which carry seven-point mutations which disrupt PDGFR-*β* signalling, have a loss of pericytes and SMC during brain development, but only a progressive region-dependent loss of BP on cortex, hippocampus, and striatum after birth; SMC populations were not affected at the time when pericyte loss was established [286]. These data suggest that PDGFR-*β* signalling is important to define the vascular phenotype, when BP are important components and SMC involvement is not strictly necessary.

#### 4.2.3. Liver

In the liver, MMC are also found in different compartments, namely, surrounding the central and portal veins and hepatic artery branches, and in a specialised form known as hepatic stellate cells (HStC), which are located in the perisinusoidal space, between fenestrated liver endothelium and epithelial hepatocytes. HStC are involved in biological roles such as blood flow regulation, by modulating cellular contraction, and immunoregulatory properties, by enhancing differentiation and accumulation of regulatory T cells [287, 288]. HStC are identified by their expression of desmin, glial fibrillary acidic protein (GFAP) when they are in a quiescent state; however in activated state they change their phenotype and express *α*-SMA [289–291].

In pathological contexts, such as liver injured, viral infections, or hepatic toxins, HStC receive signals from hepatocytes and immune cells, and they transdifferentiate in myofibroblasts. Then they secrete cytokines and growth factors responsible for promoting liver regeneration either directly by the enhancement of liver progenitor cells and hepatocytes proliferation or indirectly by endothelial cells and immune cells [292]. Activated HStC proliferate and produce extracellular matrix, inducing fibrosis in response to the liver damage, and PDGF is the most important growth factor involved in their proliferation* in vitro*. In addition, VEGF is released by HStC during liver injury and corroborates neovascularisation and organ repair [293].

#### 4.2.4. Lungs

In lung vascular beds, pericytes are located on the abluminal side of pulmonary microvessels and in part of the ABB and are distinguished from other lung mesenchymal cells by their PDGFR-*β* and NG2 expression. Lung pericytes (LP) are involved in the vascular permeability regulation and remodelling of the extracellular matrix (ECM); besides, they play important roles during the lung development, homeostasis, and response to injury and pathogens from ABB, as well as recovery from damage [294]. As well as in the liver, LP adopt a myofibroblasts phenotype in a fibrogenic microenvironment [295]. In accordance with this background information, Sava* and colleagues *[296] demonstrate that LP show a phenotypic transition in response to direct contact with ECM in the fibrotic human lung. Since isolated PC cultured on decellularized matrices from fibrotic lungs adopt expression of *α*-SMA and the administration of an inhibitor of lung fibrosis restores the elastic component of these fibrotic matrices by reverting the *α*-SMA LP phenotype, this opens a new field of study and reveals targets for pulmonary fibrosis treatment.

#### 4.2.5. Bone Marrow

Vascular niche related to BMBB is characterized by different perivascular populations and bone marrow pericytes (BMP) can be find in different compartments expressing a sort of markers [297]. Different BMP present distinct immunophenotypic profile, as follows: perisinusoidal pericytes were characterized by the expression of CD45^+^/CD146^+^ [298], Lin^−^/CD271^−^/CD45^−^/CD146^+^ [299] and CD146^+^/LepR^+^/vWF^−^/CD45^−^/CXCL-12^+^ [300]; periarteriolar pericytes were characterized by CD45^−^/Nestin^+^ [301] and CD45^−^/Nestin^+^/NG2^+^ [302]; and pericytes associated with the intima layer are Nestin^+^/CD31^+^/CD105^+^/CD90^−^/CD73^−^/CD271^−^ [303]. It is important to emphasize that the cells characterized as pericytes in these studies presented a mesenchymal activity and more studies are necessary to confirm if they are indeed pericytes derived from MSC in BM niches. However, some authors have emphasized that the expression of pericyte-related markers and cell position are sufficient to characterize and define a pericyte population and that the similarities to MSC led to the hypothesis that some subsets of MSC could act as pericytes [304–306].

BMP are linked to a multipotent lineage commitment, showing the ability to differentiate in cells of bone, adipocyte, fibroblast, and blood capillary cells in an immunodeficient mice model [299], to promote chondrogenic regeneration, and to improve angiogenesis [307]. They have an additional regenerative propriety attributed to their capacity of favouring the reconstitution of the vascular niche, by a mechanism that includes increased levels of CXCL-12 [308]. Furthermore, these findings support the idea of using BMP in procedures of regenerative medicine and practical applications on this clinical field.

#### 4.2.6. Skin

In SBB, skin pericytes (SP) are located adjacent to the proliferative basal layer of the capillary network skin epidermis. In this vascular bed, they present a fusiform cell body and lateral projections with an undulated appearance and they are paralleled to the capillary course and aligned with the micro blood vessels, besides exhibiting fenestrations, essential to their role in the SBB and cell contraction [309]. SP call attention by their capacity to promote epithelial proliferation and differentiation, ECM microenvironment modification, and tissue regeneration in the absence of angiogenesis as described by Paquet-Fifield and colleagues[310]. Moreover, myeloid progenitors seem to contribute to pericyte development in embryonic skin vasculature in a mechanism TGF-*β* signalling-dependent [311].

Skin wound healing occurs with pericyte participation and they play a range of roles in this process such as angiogenic modulation, paracrine effects, and stem cell contribution to tissue [312]. During skin injury, the coagulation cascade is activated and platelets release PDGF and TGF-*β*, activating SP detached from blood vessels and initiating a physiological process of wound healing. Therefore, pericytes act as a modulator of inflammatory cells, inducing the expression of adhesion molecules (ICAM-1, VCAM-1), chemokines (CXCL-1, CXCL-8), and proinflammatory molecules (TNFR-1, TNFR-2, IL-1R, TLRs, Nod-like receptors) promoting the trafficking of inflammatory cells into the site of injury [313–315]. Inflammatory cells promote the clearance of the damaged tissue and increase of growth factors levels that stimulate neovascularisation by overexpression of Ang-1 and angiogenesis. The new blood vessels formed are important to nourish the inflamed site and this vascular stabilisation is also a SP role by cell-cell interactions and paracrine signals, besides increased TGF-*β* levels that stimulate collagen-I deposition and scar formation [316]. Furthermore, SP secrete paracrine effectors controlling the functions of parenchyma cells, contributing to the full healing of the damaged skin. SP dysfunctions, on the other hand, are related to excessive fibrosis, resulting in hypertrophic scars and keloids; the associated microvessels are occluded by an excess of pericytes and endothelial cells surrounding them, having the former cells a myofibroblast phenotype. Conversely, SP also regulate the anoikis of endothelial cells that are not necessary by CXCR-3 activation [317, 318].

#### 4.2.7. Tumour

Regarding the tumoural microenvironment, a disorganised vasculature is observed with abundant or insufficient coverage of mural cells, depending on the site and type of tumour. Tumour pericytes (TP) are poorly attached to the vascular endothelium and present cytoplasmic projections invading the tumour parenchyma [319, 320]. Mural cells also compose a heterogeneous population on tumoural sites which present decreased EC attachment, leading to an unstable and proliferative phenotype, as well as a concomitant loss of gap junctions and cell-cell communication [321]. In concordance with this, Ribeiro and colleagues [320] had demonstrated that TP express a distinct immunophenotypic profile compared to pericytes from normal tissues and also in different tumoural microenvironments. TP isolated from childhood ependymoma and neuroblastoma specimens displayed angiogenic characteristics and expressed typical markers, such as CD146, NG2, CD90, and PDGFR-*β*. The major difference was observed in the percentage of expression by different tumour cells, differences which, in turn, could be in the tumour behaviour and its tendency to metastasise.

Several agents are related to the capacity of MMC to induce tumour progression. In this sense, VEGF shows an important contribution to induce tumoural progression, since this growth factor seems to destabilize vascular integrity by interrupting VE-cadherin function in EC and their ablation leads to an increasing of mural cells coverage and tumourigenesis acceleration [322, 323]. Recent findings reveal that lysyl oxidases (LOX) and their ligands LOX-like 1-4 (LOXL-l-4) have been implicated in vascular remodelling during tumoural angiogenesis, and LOX family constituents were detected in TP [324, 325]. LOX enzymes presented modulatory effects on activated pericytes, implicated in tumour progression evidenced by an increased migration, proliferation, and angiogenesis in ependymoma and neuroblastoma. In addition, LOX/LOX-l inhibitors could be an important target to control these types of tumour. Pericytes are also involved in dysfunctional vessel formation in gliomas. TP from rat RG2 gliomas are morphologically abnormal and desmin-positive and express heat-shock protein 47, an important modulator in the basement membrane formation, which suggests that RG2 pericytes promote angiogenesis by enhanced basement membrane production, preventing functional abnormalities in the BBB [326].

It is known that some tumours are resistant to vascular disrupting agents (VDAs) during antitumourigenic therapies, and pericytes are involved in this resistance due to the high coverage around tumoural blood vessels. Agents able to disrupt tumour peripheral vessels and become the rim penetrable are promising to avoid VDA resistance. Chen and colleagues [327] developed a prodrug, namely, Z-GP-DAVLBH, which can be selectively activated by fibroblast activation protein *α* (FAP*α*) in TP, destroying the cytoskeleton of FAP*α*-expressing TP, promoting blood vessels disruption, and turning the tumour more responsive to VDA treatments. MMC lose the expression of traditional SMC/pericyte markers in response to tumour secreted factors, increasing cell proliferation and migration, and ECM deposition. Besides, MMC in a tumour microenvironment become less undifferentiated, which causes a higher predisposition to metastasis by enhancement of ECM production, which promotes a fibronectin enrichment and creates a prometastatic niche [328, 329]. Still on promising antitumoural strategies based on MMC involvement, Murgai and colleagues [329] studied the role of pluripotency gene KLF-4 in inducing an undifferentiated phenotype in MMC. By genetic inactivation of KLF-4 in MMC, they were able to decrease prometastatic niche formation and metastasis in orthotopic metastatic B16-F10 melanoma and metastatic M3-9M rhabdomyosarcoma in mice. These data suggest a potent new approach for limiting metastasis by a strategy involving MMC modulation.

To summarize, MMC constitute a very plastic population of cells and they are able to modify their phenotype and physiological activities in accord with the tissue microenvironment. Pericytes and SMC are involved also in the pathogenesis of diseases in the majority of the organs, either by their dysfunction or by excessive activation of these cells. Therefore, understanding the mechanisms by which MMC help developing pathologies becomes a crucial step in order to propose strategies and treatments with regard to microvascular diseases in different organs and tissues.

## 5. Molecular Heterogeneity

**Figure 6:**
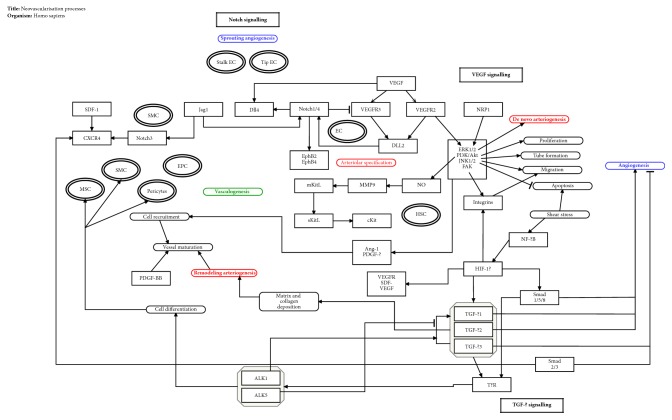
**Main signalling pathways coordinating the diversity of vessel formation processes. **NOTCH, VEGF, and TGF-*β* molecular cascades interact, by triggering and/or repressing one or more cellular functions and processes of neovascularisation. EC: endothelial cell. SMC: smooth muscle cell. MSC: mesenchymal stem cell. EPC: endothelial progenitor cell. SDF-1: stromal cell-derived factor 1. VEGF: vascular endothelial growth factor. CXCR4: C-X-C motif chemokine receptor 4. Jag1: jagged 1. Dll4: delta-like 4. VEGFR2/3: vascular endothelial growth factor receptor 2/3. NRP1: neuropilin-1. EphB2/4: ephrin B2/4. Dll2: delta-like 2. ERK1/2: extracellular signal-regulated kinase 1/2. PI3K: phosphoinositide 3-kinase. Akt: protein-serine/threonine kinase. JNK1/2: c-Jun N-terminal kinase 1/2. FAK: focal adhesion kinase. NO: nitric oxide. MMP9: matrix metalloproteinase 9. mKitL: membrane stem cell factor ligand. sKitL: soluble stem cell factor ligand. c-Kit: stem cell factor receptor. HSC: haematopoietic stem cell. Ang-1: angiopoietin 1. PDGF-*β*: platelet-derived growth factor receptor beta. HIF-1*α*: hypoxia-induced factor alpha. NF-*κ*B: nuclear factor kappa B. PDGF-BB: platelet-derived growth factor BB. TGF-*β*1/2/3: transforming growth factor beta 1/2/3. T*β*R: transforming growth factor receptor. ALK1/5: activin receptor-like kinase 1/5. The pathway has been deposited on the open repository WikiPathways, available for consultation and further improvement on https://www.wikipathways.org/index.php/Pathway:WP4331.

In addition to the heterogeneity in the neovascularisation processes and cellular level, differences regarding the molecular control are also observed. In this topic, the heterogeneity in the context of the molecular control of the microvasculature will be addressed considering the main signalling pathways that modulate the different processes of vessel formation. The heterogeneity of cells, mediators, and signalling pathways reflect the complexity in controlling the endothelium integrity and its ability to properly respond to physiological and pathological stimuli (Figure 6).

In this context, the neovascularisation processes are initiated through release of growth factors in response to either tissue hypoxia, alterations on metabolic demand or shear stress, injury, or pathological processes. For that, endothelial, mural, and inflammatory cells act together for the maintenance of the perfusion and physiological microvascular functions. Each of these cell types is intrinsically associated with the different vessel formation processes, through activation of different signalling pathways depending on the physiopathological context, i.e., the microenvironment where they are included. Although there are several molecular pathways involved, we will highlight the three major signalling pathways that regulate the neovascularisation processes, namely, the NOTCH, VEGF, and TGF-*β* pathways.

### 5.1. NOTCH Signalling Pathway

In the vascular system, NOTCH signalling is important both in determining the fate of cells and for the maintenance of vascular cells. Progenitor endothelial cells, in which NOTCH signalling is present, exhibit arterial speciation detrimental to the vascular phenotype; thus, this signalling pathway is responsible for generating a phenotypic heterogeneity in EC. On cell maintenance, EC receptors interact with smooth muscle cell ligands trough NOTCH signalling. Conversely, without NOTCH signalling, separation of EC and smooth muscle cells occurs [330]. In addition, NOTCH signalling mediates vessel maturation and stabilisation, by promoting endothelial, smooth muscle, and mural cells recruitment and proliferation, with this signalling becoming essential to neovascularisation processes. All these functions follow delta-like ligand (Dll) and jagged (Jag) signalling, which bind to NOTCH1/4 receptors. During sprouting angiogenesis, NOTCH signalling is responsible for promoting tip or stalk cell speciation. DLL-4 positive tip cells extend numerous filopodia while Jag1 positive stalk cells proliferate to form the capillary. However, at the same time when VEGF upregulates DLL-4 in tip cells, it activates NOTCH in stalk cells. Activation of NOTCH in the downstream of stalk cells regulates VEGFR-2 and VEGFR-3 expression, suppressing the tip cell phenotype in stalk cells. Pericytes and smooth muscle cells expressing NOTCH-3 are recruited to stabilise vessels in a Jag1 dependent process [331].

NOTCH is also important during the arteriogenesis process, promoting arteriolar speciation. In this case, expression of angiogenic factors such as VEGF and FGF enhances the expression of Dll2 in EC, inducing the expression of EPHB-2 by NOTCH activation. Additionally, the activation of EPHB-4 in arterial EC leads to vascular morphogenesis, contributing to remodelling arteriogenesis. Consonantly, interactions between Jag1 ligands and NOTCH-1/4 receptors lead to the transcription of genes also promoting remodelling and arterial fate speciation in neoformed arterioles. Finally, NOTCH was shown to positively modulate the expression of C-X-C chemokine receptor type 4 (CXCR-4), the receptor for CXCL-12/SDF-1, which has significant importance in vasculogenesis. Since CXCR-4/CXCL-12 signalling leads to increased mobilisation, recruitment, and roaming of putative endothelial progenitor cells (EPC), pericytes, and muscle progenitor cells, NOTCH indirectly regulates vasculogenesis. In fact, EPC stimulated by NOTCH ligands and transplanted to mice submitted to hindlimb ischaemia presented recovery of blood flow [332].

In short, NOTCH signalling pathway influences the neovascularisation processes, such as sprouting angiogenesis, arteriogenesis, and vasculogenesis, in a different way. In angiogenesis, NOTCH is important to initiation of the sprouting and vessel stabilisation, whereas in arteriogenesis it is responsible for inducing arteriolar speciation and, in vasculogenesis, NOTCH participates in the modulation of CXCR-4/CXCL-12 recruitment signalling. Thus, the NOTCH signalling exhibits a heterogeneous biological role in different neovascularisation forms.

### 5.2. VEGF Signalling Pathway

VEGF signalling is another important pathway to the microvascular and neovascularisation control, participating in vascular homeostasis and protection. It is known that VEGF promotes EC survival, since increased VEGF levels inhibit cell apoptosis by phosphoinositide 3 kinase (PI3K) and antiapoptotic kinase (AKT) activation. In addition, VEGF induces EC migration, stimulating angiogenesis by focal adhesion kinase (FAK) activation and promoting integrin interactions [333].

In fact, VEGF appears to exert specifics and significative effects in each different process of neovascularisation. In general, the activation of VEGFR-2 leads to increased sprouting angiogenesis. In this case, VEGF-A-mediated signalling leads to signal amplification, with increased expression of ERK1/2, PI3K, JNK1/2, and FAK. The MAPKs pathway induces tube formation while AKT leads to NO production and cell proliferation. At the same time, JNK1/2 activation leads to increased cell migration. Together, all these effects induce increasement of angiogenesis. Besides that, VEGF also collaborates with sprouting angiogenesis by initiating tip and stalk cell phenotypes along with NOTCH signalling, as mentioned before. It was demonstrated that tip cells express more VEGFR-2 and are therefore more responsive to VEGF ligands, while stalk cells express less VEGFR-2 but still proliferate more. These characteristics allow sprouting process to occur toward the VEGF gradient followed by tip cells. Vessel stabilisation also occurs through participation of VEGF, which initiates vascular maturation by stimulating PDGF-B and angiopoietin-1 (Ang-1) expression, growth factors responsible for the recruitment of mural cells, such as pericytes and smooth muscle cells [334, 335].

The effects of VEGF signalling on arteriogenesis have been also investigated. It is known that the heterodimerisation of VEGFR-2 and NRP-1 induces ERK expression and, consequently, stimulates de novo arteriogenesis. Additionally, shear stress in arteries and arterioles leads to the activation of proinflammatory pathways, such as of NF-*κ*B, which culminates with HIF-1*α* production and higher expression of cell adhesion molecules and VEGF receptors, essential for remodelling arteriogenesis. Moreover, VEGFR-2/NRP-1 signalling leads to capillary arterialisation, lumen expansion, and vascular remodelling which, concomitantly to PDGF-B activation, leads to maturation and stabilisation of vessels due to the recruitment of mural cells, completing the arteriogenesis process [336].

In vasculogenesis, especially after ischaemic events, VEGF and CXCL-12 are released in response to HIF-1*α* higher expression, stimulating NO release by EC. NO in turn stimulates MMP9 activity, which leads to cleavage of membrane-bound stem cell factor (mKitL) and release of the soluble factor (sKitL). The interactions between sKitL and the haematopoietic stem cell surface receptor (c-Kit) lead to mobilisation of angiogenic stem cells from the vascular niche on bone marrow to bloodstream. In this way, the release of putative EPC from bone marrow to the bloodstream and their recruitment to peripheral ischaemic tissues occur in response to chemoattractant gradients, including VEGF and CXCL-12. In addition, these soluble factors initiate the committing of these stem cells with the endothelial lineage, ultimately inducing its differentiation into mature EC and promoting the revascularisation of the ischaemic area [336]. Therefore, VEGF signalling seems to be important in different ways by inducing the expression of distinct molecules in the cells associated with the microvasculature.

### 5.3. TGF-*β* Signalling Pathway

Another signalling pathway closely involved in neovascularisation processes is from that of tumour growth factor *β* (TGF-*β*). In the vascular system, TGF-*β* mediates several biological functions related to vascular remodelling, such as matrix and collagen deposition, EC chemotaxis, and either inhibition or stimulation proliferation of EC, as well as regulation of vasoactive peptides and growth factor release [337]. In hypoxic conditions, the bioactivation of TGF-*β*2 and its coreceptors from the Smad family are stimulated by HIF-1*α*, which stimulates an increasing in TGF-*β*2 transcription. Additionally, hypoxia generates oxidative stress that triggers profibrogenic pathways, among which is TGF-*β* signalling, thus generating collagen deposition and vascular remodelling [338].

In general, TGF-*β* pathway has a stimulatory effect on the angiogenesis process. In a quiescent state of EC, TGF-*β*/TGF-*β* receptor signalling is inhibited and cell proliferation does not occur. However, a proangiogenic stimulus is able to activate this pathway and induce the proliferation and migration of EC. Interestingly, TGF-*β* signalling also can mediate antiangiogenic responses, depending on which coreceptors are activated. The activation of Smad 1, 5, and 8 induces increased proliferation, migration, and matrix degradation, while activation of Smad 2 and 3 culminates in reduced angiogenesis. Besides, TGF-*β*1 and TGF-*β*2 are known for exerting a stimulatory effect on angiogenesis and TGF-*β*3 an inhibitory one, despite the fact that TGF-*β*3 can also stimulate specific actions such as reepithelialisation [339, 340]. Vessel maturation and stabilisation are also TGF-*β* signalling-dependent, once it promotes differentiation of pericytes. This effect is due to the balance between the signalling exerted by T*β*R/ALK-1 and that by T*β*R/ALK-5, which play, respectively, a positive and a negative modulation on TGF-*β* synthesis in EC.

TGF-*β* also is involved in arteriogenesis and vasculogenesis processes. Pathological shear stress triggers endothelium activation and apoptosis of EC, stimulating the migration of macrophages into the interstitial space. Increased TGF-*β* production by macrophages induces vascular remodelling and differentiation of myofibroblasts into smooth muscle cells, a crucial step to the stabilisation of new arterioles. In the vasculogenesis context, the role of TGF-*β* relates to the fact that TGF-*β* signalling pathway acts on both haemangioblasts and mesenchymal stem cells inducing their differentiation. Interestingly, it was demonstrated that TGF-*β* promotes haematopoietic stem cells differentiation in mice, contributing to the formation of new vessels by haematopoietic precursors in a cutaneous wound healing model. In addition, TGF-*β* upregulates CXCR-4 expression by a Smad 2/3-dependent mechanism, also favouring the recruitment of bone marrow cells [341, 342]. Thus, a range of distinct biological effects, modulating and influencing directly the processes of neovascularisation, is observed.

Overall, in spite of the existence of heterogeneity related to the molecular control of the neovascularisation processes, this heterogeneity occurs in a more restricted way when compared to the inter and intracellular heterogeneities related to the different neovascularisation processes. Interestingly, this restricted heterogeneity allows the general control of the biological processes of the microvasculature to be exerted in a punctual form by reduced signalling pathways that converge, although they receive inputs of components and processes that exhibit great heterogeneity.

## 6. Final Considerations

The neovascularisation processes—vasculogenesis, angiogenesis, arteriogenesis, and lymphangiogenesis—are controlled by specific factors released by several cell types, including endothelial, mural, and inflammatory cells, and they are essential for organ and tissue development and homeostasis throughout life. The range of factors and cell types involved in these processes reflects the different metabolic needs and organotypic functions in different vascular beds. In this sense, the different blood-organ barriers are important components in the maintenance of tissue homeostasis, since they are responsible for the exchange and direct communication between different tissue compartments, acting in the control of the flow and influx of molecules, biological factors, and cell trafficking. These barriers are part of the tissue microvasculature, and endothelial and murals cells are essential for coordinating their functions. In fact, any alteration in the cellular constituents can lead to microvascular changes, altering patterns of molecule expression, which may implicate alterations in the physiology of the different types of neovascularisation. The better understanding of the heterogeneity related to the mechanisms, cell types, and molecular factors involved in the different neovascularisation processes provides new perspectives for the delineation of therapeutic and pharmacological strategies that may limit or control changes associated with the microvasculature. In addition, understanding the cell and molecular heterogeneity through which different neovascularisation processes occur would allow the identification of potential specific therapeutic targets and biomarkers that can act in the context of the diversity of microvascular beds and related diseases.

Finally, we believe that the understanding of the heterogeneity involved with the microcirculation is fundamental for the solid and integrated construction of knowledge about the biology of the vascular system. Studying the aspects of heterogeneity in isolation can generate a simplistic viewpoint of the physiological and pathological processes that occur in a microvascular context. Therefore, the cellular heterogeneity and the heterogeneity associated with the molecular control and with the different forms of neovascularisation complement each other in order to promote a functional microenvironment which responds in a complex way to alterations that could disturb its homeostasis.
